# Supplementary material for: TCTP regulates genotoxic stress and tumorigenicity via intercellular vesicular signaling
Source: EMBO Rep. 2024 Mar 28;25(4):20. doi: 10.1038/s44319-024-00108-7 (PMC11014985; doi:10.1038/s44319-024-00108-7)
Supplement: Supplementary file 1 — Appendix [file 44319_2024_108_MOESM1_ESM.pdf]

Appendix

Table of content

Appendix Figure S1 **Page 2**

Additional information for the generation of *Tctp*$^{-/f-}$ mice.

Appendix Figure S2 **Page 8**

Analysis of the protein interaction between TSAP6 and TCTP and effect of sertraline and thioridazine on the complex formation.

Appendix Figure S3 **Page 10**

Uptake by sh7239 MDA-MB231 cells of FITC labeled sEVs derived from the parental MDA-MB231 cells.

Appendix Figure S4 **Page 11**

Uptake of FITC labeled sEVs derived from sh7239 MCF7 cells by sh7239 MCF7 cells.

Appendix Figure S5 **Page 12**

Uptake of FITC labeled sEVs derived from sh7239 MDA-MB231 cells by sh7239 MDA-MB231 cells.

Appendix Figure S6 **Page 13**
Effect of sEVs on TCTP expression.

A

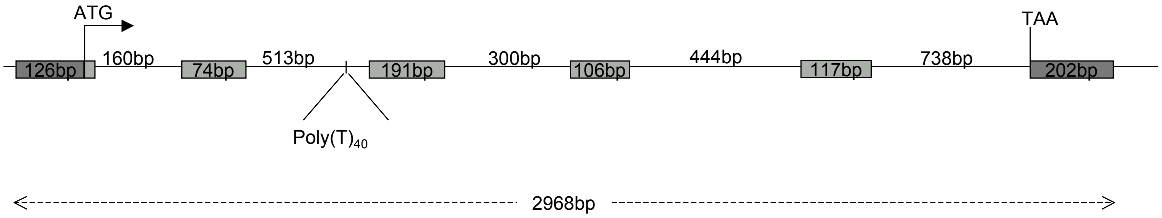

NC_000080 Mus musculus chromosome 14, reference assembly (C57BL/6J)

B

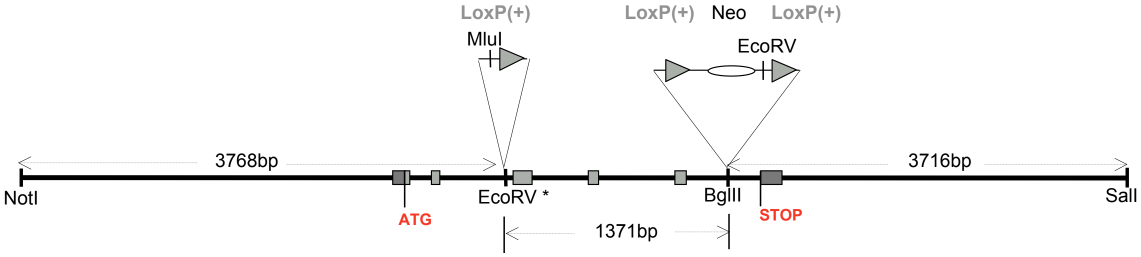

* Eco RV: site added in the construct

C

uncut	circular
Hind III	13431bp
Sal I	13431bp
Not I	13431bp
MluI	13431bp
Not I + Sal I	2811bp + 10620bp
Eco RV	46bp + 2983bp + 10402bp
BglII	1732bp + 11699bp

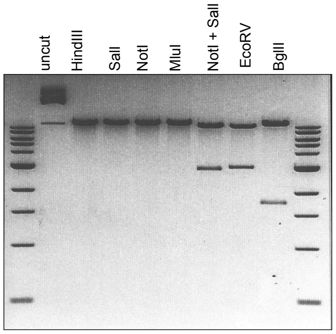

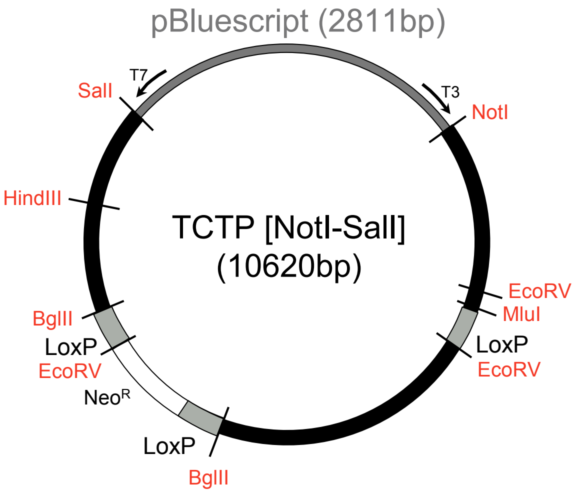

D

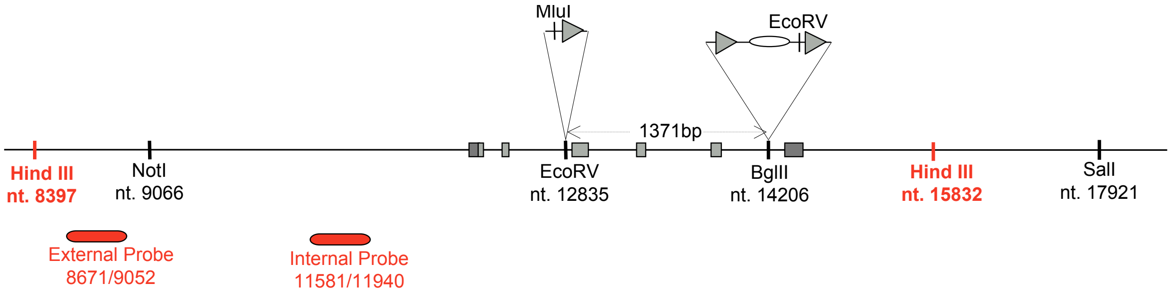

	DIGESTION	wild type	TCTP (L3)	TCTP (L2)	TCTP (L-)
Internal probe	Hind III	7435bp	9253bp	7483bp	6054bp
External probe	Hind III	7435bp	9253bp	7483bp	6054bp

External probe:
aagcaggccattgagcaacttcctgctcagggaaaa
tcagctgatctgccagccccatccacaagggctggct
tgaggctttctctcttcagcacctgcactttgaaagggt
atcaagttctttcttttggttactattttggtctctctcaacct
ggctgcacatggacattctctgaagatttaaaaaact
cagatgcctggatcccacccccagaatttctaactcta
ctgaacttgtgtgccggcttgacatcatttttcccctca
agttccgactgccaggcaggtggactacagatctatt
gacctctagactgttcaccactgtcaatgtgactttaac
agccctcaatattaagttagagctcagctcaccac

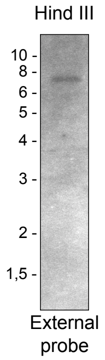

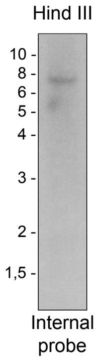

Internal probe:
ctccgcgaacaagacgggtgcagtctgtgggcagc
caaggaccggagatcggagacccgggacaccag
cccggcgccatccccgtagtccccgcaggctcagc
ggcgcgcccgacaacagtcatccgattctgctgaca
ttttctttccgagaaaggggtgggaactgaagcggtgt
ggcgggaggcggggcgcagccacaccctggcca
cgcccgggcggcgactcaagcgtccggccatcggt
cggccgcaagtcccttcccgtcccagcatgccccgg
gcgcactatccgcacaccgcccccgttgccccgcgc
acccaggggcactccgcattgtgtcccagccgagtc
cccggat

E

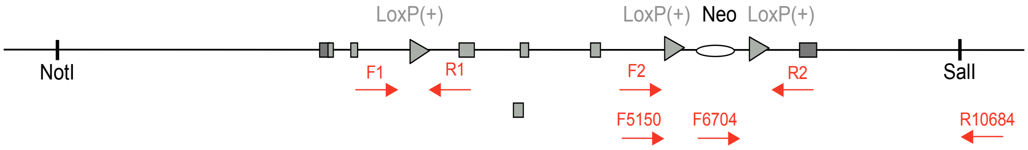

	Wild type	TCTP (L3)
**F1/R1**	292pb	344bp
**F1/R2**	1703bp	3487bp
**F2/R2**	329bp	2061bp
**F5150/R10684**	3796bp	5534bp
**F6704/R10684**	-	3980bp

Polymerase	Program
Phusion (0.2µl/25µl)	35 cycles - Tm annealing: 60°C - Extension: 1min
Expand (0.4µl/25µl)	10 cycles - Tm annealing: 60°C - Extension 6min<br>+ 20 cycles - Tm annealing 60°C - Extension 6min + 20sec/cycle
Expand (0.4µl/25µl)	
Expand (0.4µl/25µl)	
Phusion (0.2µl/25µl)	35 cycles - Tm annealing: 60°C - Extension: 4min

**F1**	5'-GAGTGACCACCTCTCTCAGTCC-3'
**R1**	5'-AACACCGGTGACTACTGTGCTTTCG-3'
**F2**	5'-GGGAGTGCAGCAGTGATTCTTTGCC-3'
**R2**	5'-AAGGAACACTGTCTCCACCAGTCCC-3'
**F5150**	5'-GTGGCAGGCCTTGTAGATTGTGAGAT-3'
**F6704**	5'-GGGTGGGGTGGGATTAGATAAATGC-3'
**R10684**	5'-GAACTGACAGAAACCCAGGACTCAATG-3'

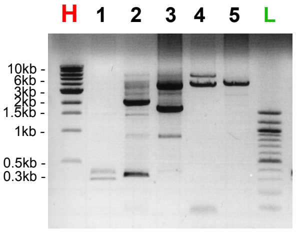

H= High molecular weight marker

PCR 1: couple F1/R1
PCR 2: couple F2/R2
PCR 3: couple F1/R2
PCR 4: couple F5150/R10684
PCR 5: couple F6704/R10684

L= Low molecular weight marker

F

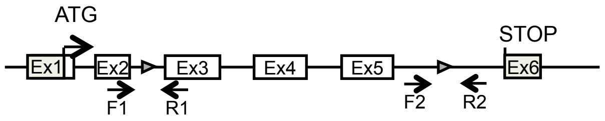

G

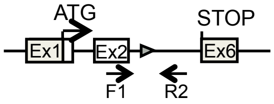

H

	WT	*Tctp* L3 lox	*Tctp* L2 lox-Neo	*Tctp* KO
**F1/R1**	292 bp	344 bp	344 bp	
**F1/R2**	1703 bp	3487 bp	1823 bp	421 bp
**F2/R2**	329 bp	2061 bp	428 bp	

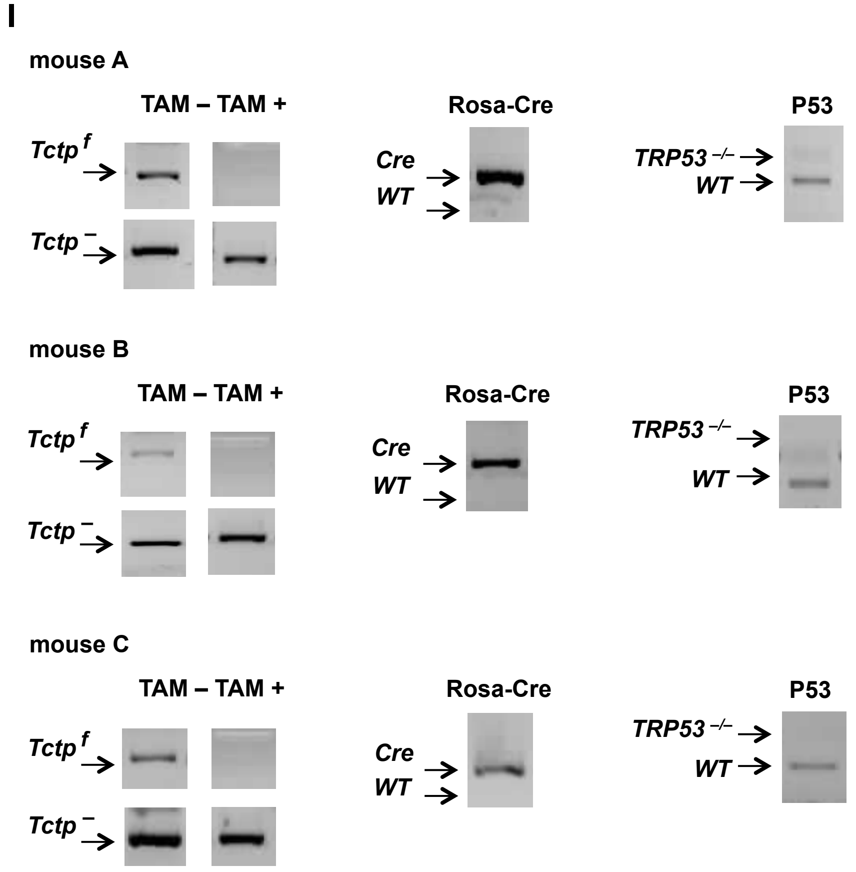
I
mouse A
TAM – TAM +
Tctp f
Tctp –
Rosa-Cre
Cre
WT
P53
TRP53 –/–
WT
mouse B
TAM – TAM +
Tctp f
Tctp –
Rosa-Cre
Cre
WT
P53
TRP53 –/–
WT
mouse C
TAM – TAM +
Tctp f
Tctp –
Rosa-Cre
Cre
WT
P53
TRP53 –/–
WT

Appendix Figure S1. Additional information for the generation of *Tctp*$^{–/f–}$ mice.

A Schematic representation of *Tctp* on chromosome 14.

B Insertion of an additional Eco RV site (*).

C Insertion of the construct in the pBluescript plasmid, with a gel showing the restriction fragments using the different enzymes and a table with their size.

D Southern blot analysis of the integrated vector after homologous recombination, using with two different probes.

E L3-construct:

For the genotyping of the ESCs (above schematic representation and PCR products) for the inducible *Tctp*-knockout allele the following primers were used:

PCR1 (F1/R1), lane 2: two bands were amplified, a band of 292 bp that corresponded to the *wild-type* (*WT*) allele and a slightly stronger band of 344 bp for the floxed allele L3.

PCR2 (F2/R2), lane 3: two main bands were amplified, one of 329 bp (*WT* allele) and 2061 bp for L3

PCR3 (F1/R2), lane 5: 1703 bp for *WT* and 3487 bp for L3

PCR4 (F5150/10684), lane 6: 3796 bp (*WT*) and 5534 bp for L3

PCR5 (F6704/10684), lane 7: 3980bp for L3 only

F *Tctp* inducible allele L2 with Neo cassette deleted

G *Tctp* constitutively deleted allele

H Upon deletion of the Neo cassette, the PCR products generated are listed below (L3 with Neo; L2 Neo deleted). In order to differentiate the genotypes between

the inducible *Tctp*-knockout allele and the constitutive *Tctp*-knockout allele, a combination of the primers F1, F2, R1 and R2 was used. Typically, the PCR using the primers F1/R2 generated a band of 421 bp for the constitutive *Tctp*-knockout allele, F2/R2 for Lox3, generated a 2061 bp band and Lox2 without the Neo-cassette generated a 428 bp band.

I Examples of PCR-genotyping in three $Trp53^{-/-}$;$Tctp^{-/f-}$ mice of conditional allele $Tctp^{f}$ before (TAM–) and after (TAM+) tamoxifen treatment (mice used: *WT,* $Trp53^{-/-}$;$Tctp^{-/f-}$ *and Rosa-Cre)*. The primers used are specified above.

Appendix Amson et al.

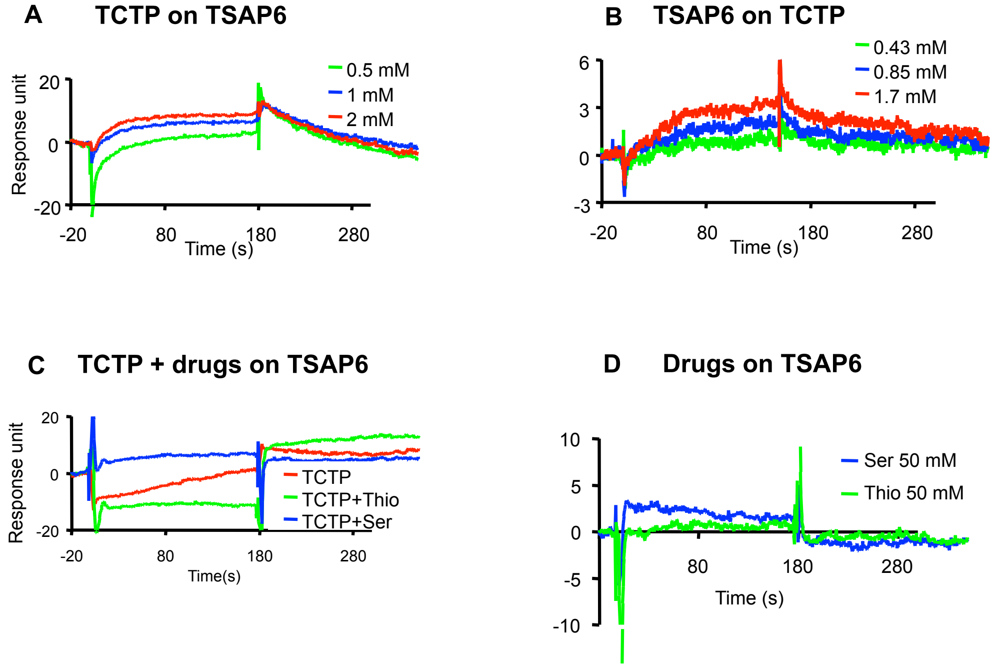

Appendix Figure S2. Analysis of the protein interaction between TSAP6 and TCTP and effect of sertraline and thioridazine on the complex formation.

A Sensorgrams of the binding of TCTP to TSAP6 immobilized, Kd: 9.52 10^{-7} M

B Sensorgrams of the binding of TCTP to TSAP6 immobilized, Kd: 9.52 10^{-7} M

The binding data for SPR experiments were fitted using a Langmuir binding model (see below)

C Sensorgrams of the binding of TCTP in presence of Sertraline and Thioridazine (100mM) to TSAP6 immobilized. The percentage of inhibition was calculated measuring the slope during the association phase: 70% of inhibition for Sertraline and 80% of inhibition for Thioridazine were obtained .

D Sensorgrams of the binding of Sertraline and Thioridazine to TSAP6 immobilized.

Data information: Langmuir binding model

(A)	ka (1/Ms)	kd (1/s)	Rmax (RU)	RI (RU)	Drift (RU/s)	Conc of ana	KA (1/M)	KD (M)	Req (RU)	kobs (1/s	Chi2
	1,85E+04	0,0176	22,3				1,05E+06	9,52E-07			0,931
TCTP				-12,5	-0,0123	2u			15,1	0,0546	
TCTP				-4,7	1,25E-03	1u			11,4	0,0361	
TCTP				1,05	4,25E-03	500n			7,67	0,0268	

(B)	ka (1/Ms)	kd (1/s)	Rmax (RU)	RI (RU)	Conc of analyte	KA (1/M)	KD (M)	Req (RU)	kobs (1/s)	Chi2
	5250	0,00475	5,03			1100000	0,000000905			0,0682
TSAP6 -0.43uM				0,00642	0.43u			1,62	0,00701	
TSAP6 -0.85uM				0,326	0.85u			2,44	0,00922	
TSAP6 -1.7uM				0,456	1.7u			3,28	0,0137	

(C)	Slope	%inhibition
TCTP	0,0768	0
TCTP+Thioridazine	0,0152	80
TCTP+Sertraline	0,0233	70

All the experiments were performed in the presence of 40 mM NaCl.

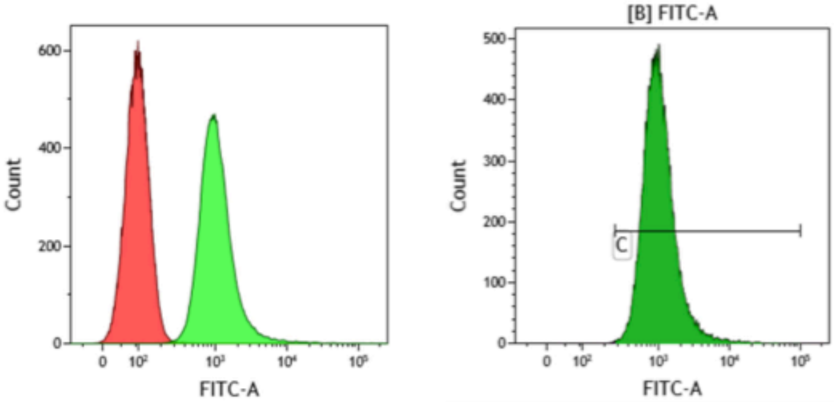

Gate	Number	%Total	%Gated	X-GMean
All	52,984	82.04	100.00	1,042.88
C	52,884	81.89	99.81	1,042.83

Appendix Figure S3. Uptake by sh7239 MDA-MB231 cells of FITC labeled sEVs derived from the parental MDA-MB231 cells.

Left graph: Unlabeled sh7239 MDA-MB231 cells (red), uptake of FITC labeled sEVs (green). Right graph: FITC positive gated population (green) (C gate). The value of the gating is displayed below the graph.

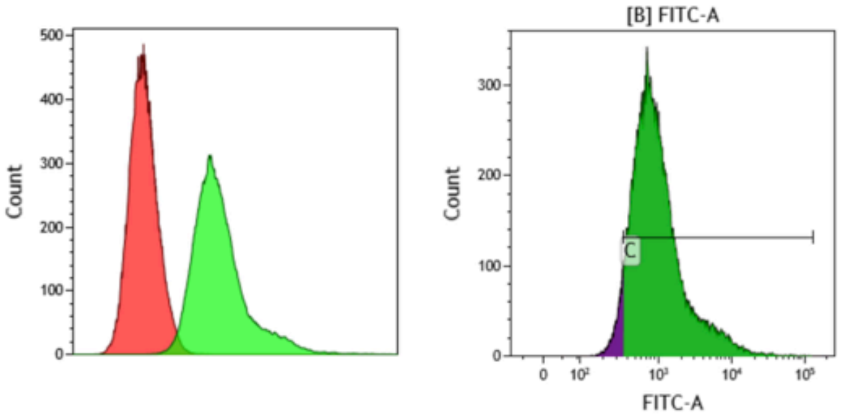

Gate	Number	%Total	%Gated	X-GMean
All	47,715	55.90	100.00	978.68
C	45,584	53.40	95.53	1,036.14

Appendix Figure S4. Uptake of FITC labeled sEVs derived from sh7239 MCF7 cells by sh7239 MCF7 cells.

Left graph: Unlabeled sh7239 MCF7 cells (red), uptake of FITC labeled sEVs (green).

Right graph: FITC negative population (purple), FITC positive gated population (green) (C gate). The value of the gating is displayed below the graph.

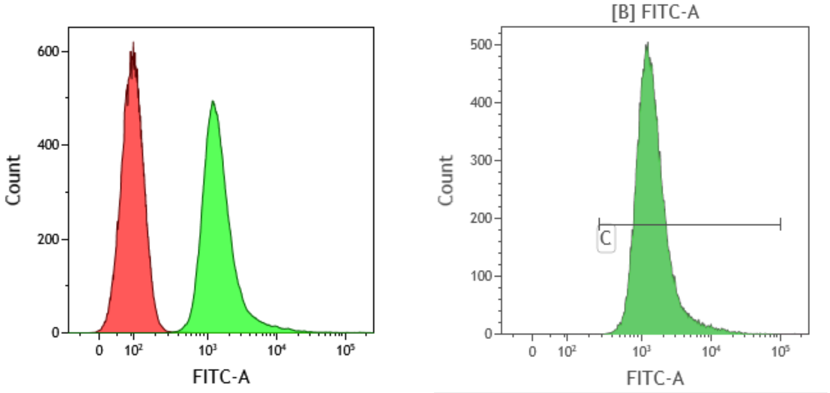

Gate	Number	%Total	%Gated	X-GMean
All	52,305	80.27	100.00	1,449.99
C	52,251	80.19	99.90	1,444.17

Appendix Figure S5. Uptake of FITC labeled sEVs derived from sh7239 MDA-MB231 cells by sh7239 MDA-MB231 cells.

Left graph: Unlabeled MDA-MB231 sh7239 cells (red), uptake of FITC labeled sEVs (green). Right graph: FITC positive gated population (green) (C gate). The value of the gating is displayed below the graph.

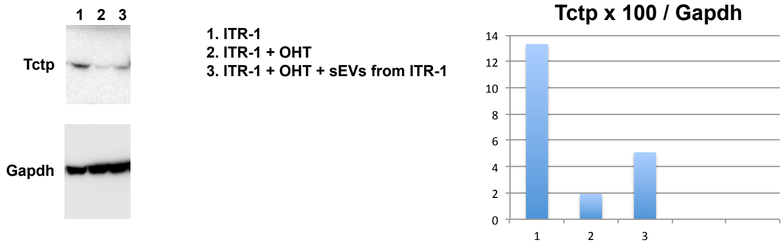

Appendix Figure S6. TCTP expression in ITR-1 cells upon supplemented with sEVs. Western blot analysis of Tctp expression in untreated ITR-1 cells (1) and in 4-OHT treated ITR-1 cells (2) supplemented with sEVs from ITR-1 cells (3). Gapdh was used as loading control. Quantification of the bands (Image Lab software from Bio-Rad).
