# Supplementary material for: TCTP regulates genotoxic stress and tumorigenicity via intercellular vesicular signaling
Source: EMBO Rep. 2024 Mar 28;25(4):20. doi: 10.1038/s44319-024-00108-7 (PMC11014985; doi:10.1038/s44319-024-00108-7)
Supplement: Supplementary file 7 — Source data Fig. 1 [file 44319_2024_108_MOESM7_ESM.zip › Source Data Figure 1/Source Data Fig 1I Left.pdf]

A

Blank: Thymocytes alone

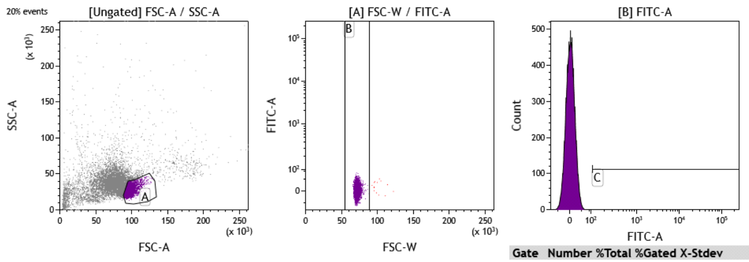

Gate	Number	%Total	%Gated	X-Stdev
All	27 837	36,39	100,00	18,12
C	0	0,00	0,00	N/A

Gate	X-GMean
All	3,19
C	N/A

B

FITC background: Thymocytes in the presence FITC

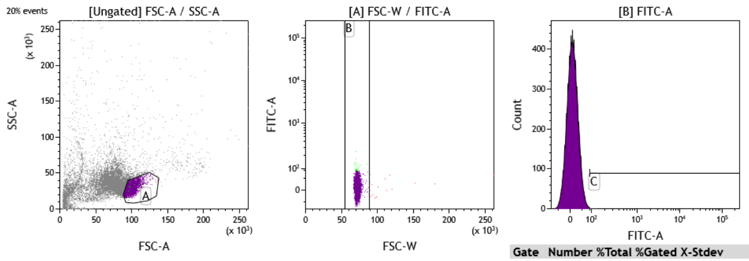

Gate	Number	%Total	%Gated	X-Stdev
All	30 367	37,42	100,00	22,84
C	28	0,03	0,09	32,52

Gate	X-GMean
All	10,12
C	111,26

C

Uptake of FITC-labeled sEVs derived from *wild type* thymocytes

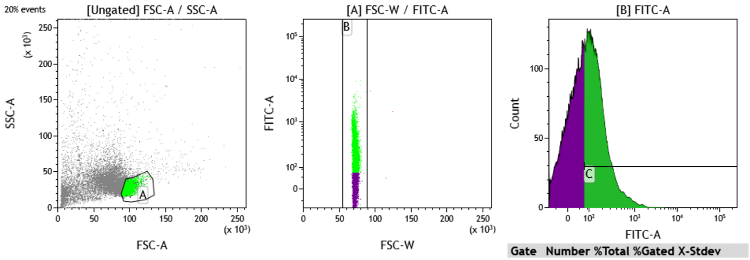

Gate	Number	%Total	%Gated	X-Stdev
All	28 124	33,20	100,00	209,80
C	15 085	17,81	53,64	249,40

Gate	X-GMean
All	80,85
C	166,25

D

Overlay blank (red) and uptake of FITC-labeled sEVs derived from *wild type* thymocytes (green)

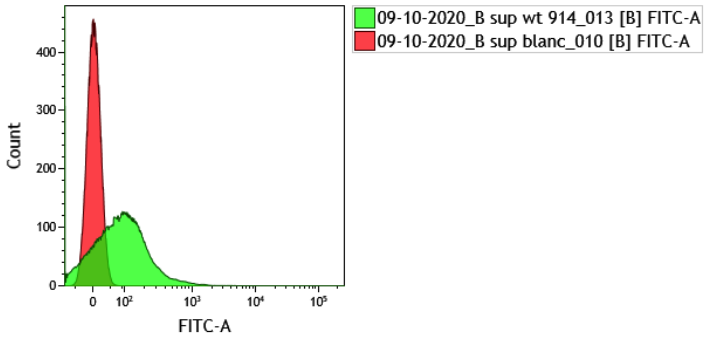

Figure 1I Left
