# Supplementary material for: TCTP regulates genotoxic stress and tumorigenicity via intercellular vesicular signaling
Source: EMBO Rep. 2024 Mar 28;25(4):20. doi: 10.1038/s44319-024-00108-7 (PMC11014985; doi:10.1038/s44319-024-00108-7)
Supplement: Supplementary file 7 — Source data Fig. 1 [file 44319_2024_108_MOESM7_ESM.zip › Source Data Figure 1/Source Data Fig 1E Left.pdf]

NANOSIGHT

WT89 1-5 SEV 2020-01-29 15-09-59

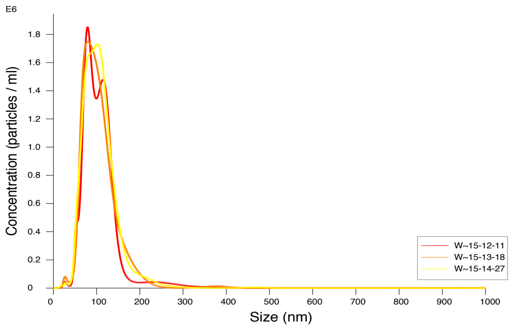

FTLA Concentration / Size graph for Experiment:
WT89 1-5 SEV 2020-01-29 15-09-59

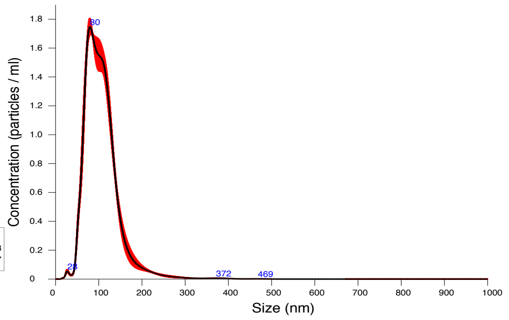

Averaged FTLA Concentration / Size for Experiment:
WT89 1-5 SEV 2020-01-29 15-09-59
Error bars indicate + / -1 standard error of the mean

Included Files

WT89 1-5 SEV 2020-01-29 15-12-11
WT89 1-5 SEV 2020-01-29 15-13-18
WT89 1-5 SEV 2020-01-29 15-14-27

Details

NTA Version:	NTA 3.3 - Sample Assistant Dev Build 3.3.203
Script Used:	SOP Standard Measurement 02-42-58PM 29J~
Time Captured:	15:09:59 29/01/2020
Operator:	WT89 1-5 SEV
Pre-treatment:	
Sample Name:	test
Diluent:	
Remarks:	

Capture Settings

Camera Type:	sCMOS
Laser Type:	Blue405
Camera Level:	16
Slider Shutter:	1300
Slider Gain:	512
FPS	25.0
Number of Frames:	1498
Temperature:	19.4 ºC
Viscosity:	(Water) 1.013 - 1.015 cP
Dilution factor:	Dilution not recorded
Syringe Pump Speed:	40

Analysis Settings

Detect Threshold:	4
Blur Size:	Auto
Max Jump Distance:	Auto: 17.8 - 19.9 pix

Results

Stats: Merged Data

Mean:	106.3 nm
Mode:	79.7 nm
SD:	41.6 nm
D10:	66.2 nm
D50:	99.9 nm
D90:	148.8 nm

Stats: Mean +/- Standard Error

Mean:	106.3 +/- 0.3 nm
Mode:	86.7 +/- 7.6 nm
SD:	41.6 +/- 0.6 nm
D10:	66.3 +/- 0.6 nm
D50:	99.9 +/- 1.0 nm
D90:	148.7 +/- 3.7 nm
Concentration (Upgrade):	1.36e+08 +/- 4.53e+06 particles/ml
	20.4 +/- 0.7 particles/frame
	23.0 +/- 0.4 centres/frame

Figure 1E Left
