# Supplementary material for: TCTP regulates genotoxic stress and tumorigenicity via intercellular vesicular signaling
Source: EMBO Rep. 2024 Mar 28;25(4):20. doi: 10.1038/s44319-024-00108-7 (PMC11014985; doi:10.1038/s44319-024-00108-7)
Supplement: Supplementary file 7 — Source data Fig. 1 [file 44319_2024_108_MOESM7_ESM.zip › Source Data Figure 1/Source Data fig 1E Right.pdf]

NANOSIGHT

KO81 1-5 SEV 2020-02-05 15-10-36

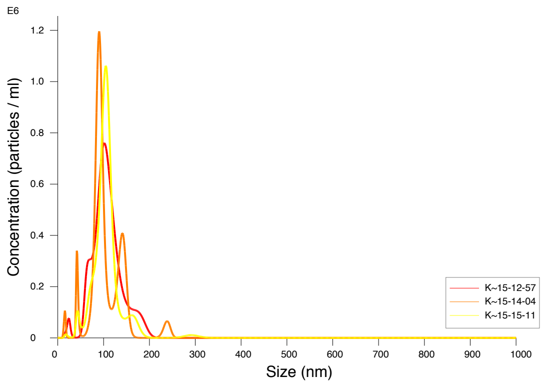

FTLA Concentration / Size graph for Experiment:
KO81 1-5 SEV 2020-02-05 15-10-36

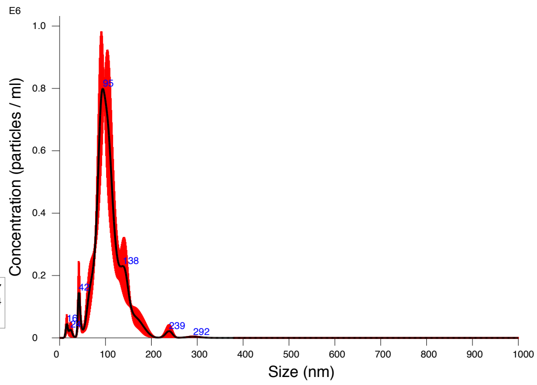

Averaged FTLA Concentration / Size for Experiment:
KO81 1-5 SEV 2020-02-05 15-10-36
Error bars indicate + / -1 standard error of the mean

Included Files

KO81 1-5 SEV 2020-02-05 15-12-57
KO81 1-5 SEV 2020-02-05 15-14-04
KO81 1-5 SEV 2020-02-05 15-15-11

Details

NTA Version:	NTA 3.3 - Sample Assistant Dev Build 3.3.203
Script Used:	SOP Standard Measurement 02-40-37PM 05~
Time Captured:	15:10:36 05/02/2020
Operator:	KO81 1-5 SEV
Pre-treatment:	
Sample Name:	KO81 1-5 SEV
Diluent:	
Remarks:	

Capture Settings

Camera Type:	sCMOS
Laser Type:	Blue405
Camera Level:	15
Slider Shutter:	1206
Slider Gain:	366
FPS	25.0
Number of Frames:	1498
Temperature:	20.3 °C
Viscosity:	(Water) 0.991 - 0.992 cP
Dilution factor:	Dilution not recorded
Syringe Pump Speed:	50

Analysis Settings

Detect Threshold:	4
Blur Size:	Auto
Max Jump Distance:	Auto: 25.6 - 33.8 pix

Results

Stats: Merged Data

Mean:	106.2 nm
Mode:	94.0 nm
SD:	33.6 nm
D10:	73.3 nm
D50:	101.5 nm
D90:	145.6 nm

Stats: Mean +/- Standard Error

Mean:	106.2 +/- 0.8 nm
Mode:	98.9 +/- 4.4 nm
SD:	33.6 +/- 1.8 nm
D10:	73.8 +/- 2.0 nm
D50:	101.1 +/- 3.2 nm
D90:	144.3 +/- 4.2 nm
Concentration (Upgrade):	4.15e+07 +/- 1.90e+06 particles/ml
	5.9 +/- 0.3 particles/frame
	9.7 +/- 0.2 centres/frame

Concentration measurements may be unreliable
See summary file for more info

Figure 1E Right
