# Supplementary figures and images for: TCTP regulates genotoxic stress and tumorigenicity via intercellular vesicular signaling
Source: EMBO Rep. 2024 Mar 28;25(4):20. doi: 10.1038/s44319-024-00108-7 (PMC11014985; doi:10.1038/s44319-024-00108-7)

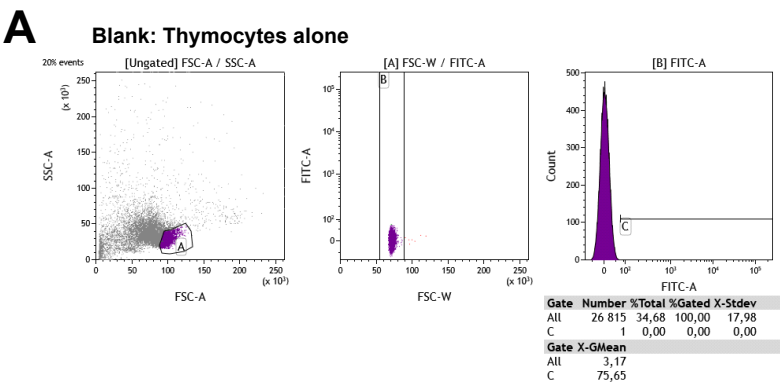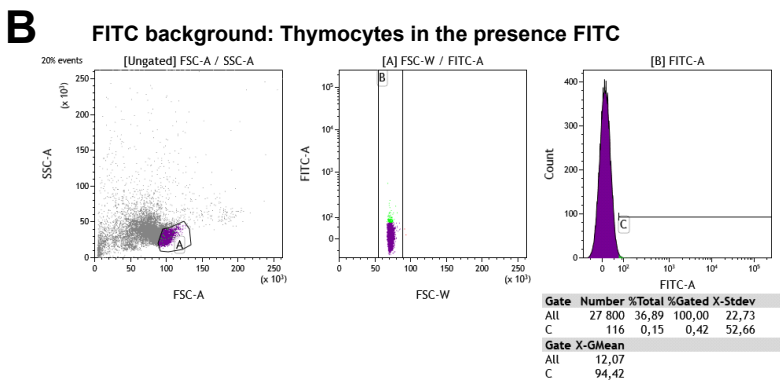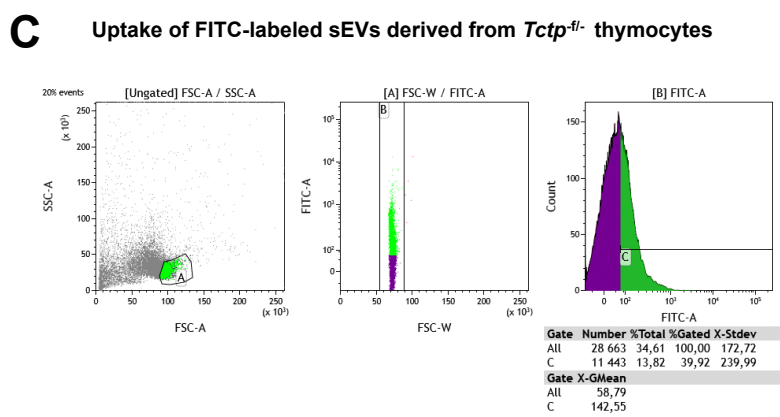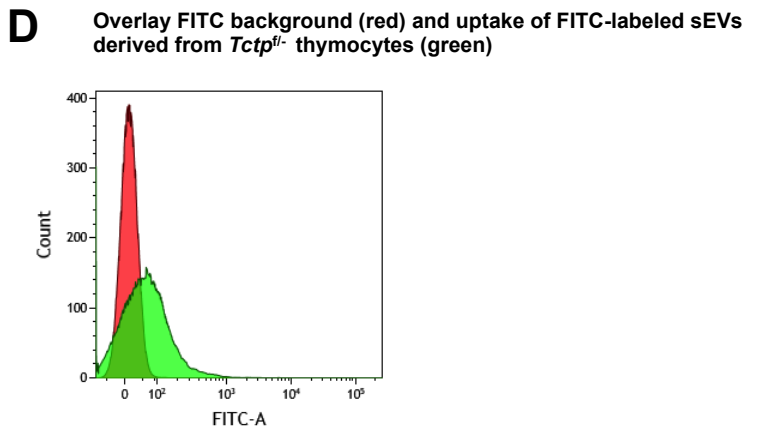

Figure 1I Right

Supplement: Supplementary file 7 — Source data Fig. 1 [file 44319_2024_108_MOESM7_ESM.zip › Source Data Figure 1/Source Data Fig 1I Right.pdf]

**Fig 1D**

**Western blots presented in the manuscript:**

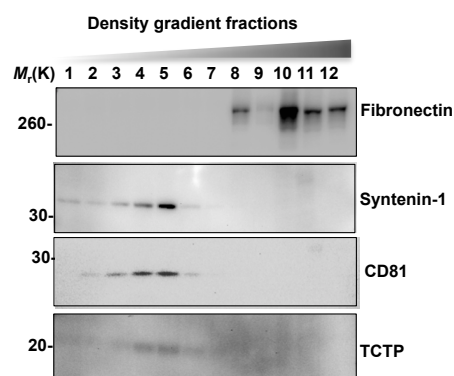

**Original uncropped Western blots:**

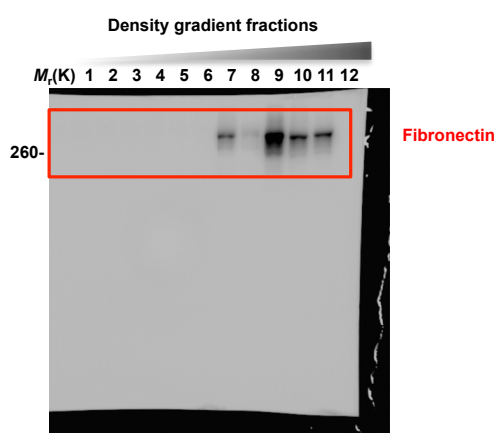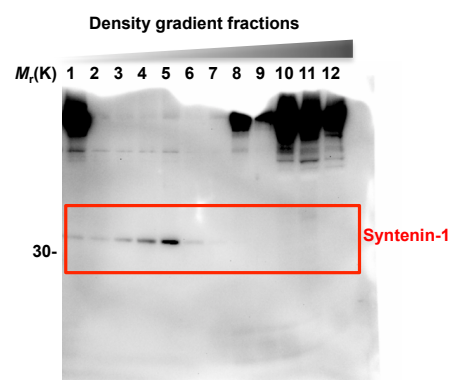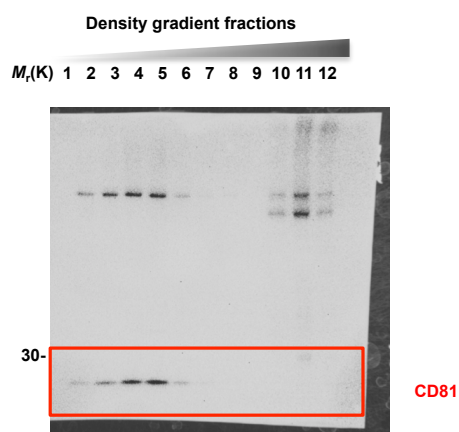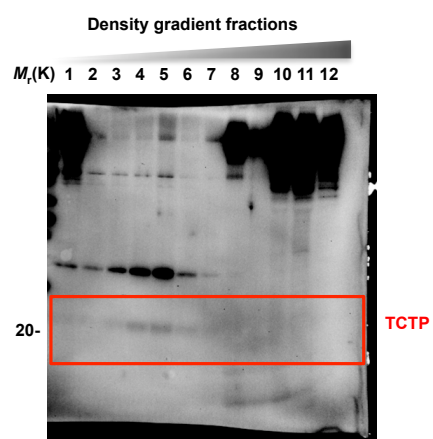

Supplement: Supplementary file 7 — Source data Fig. 1 [file 44319_2024_108_MOESM7_ESM.zip › Source Data Figure 1/Source Data Fig 1D.pdf]

Fig 1C

Western blots presented in the manuscript:

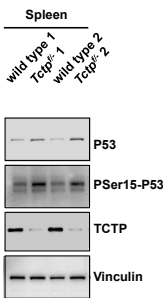

Original uncropped Western blots:

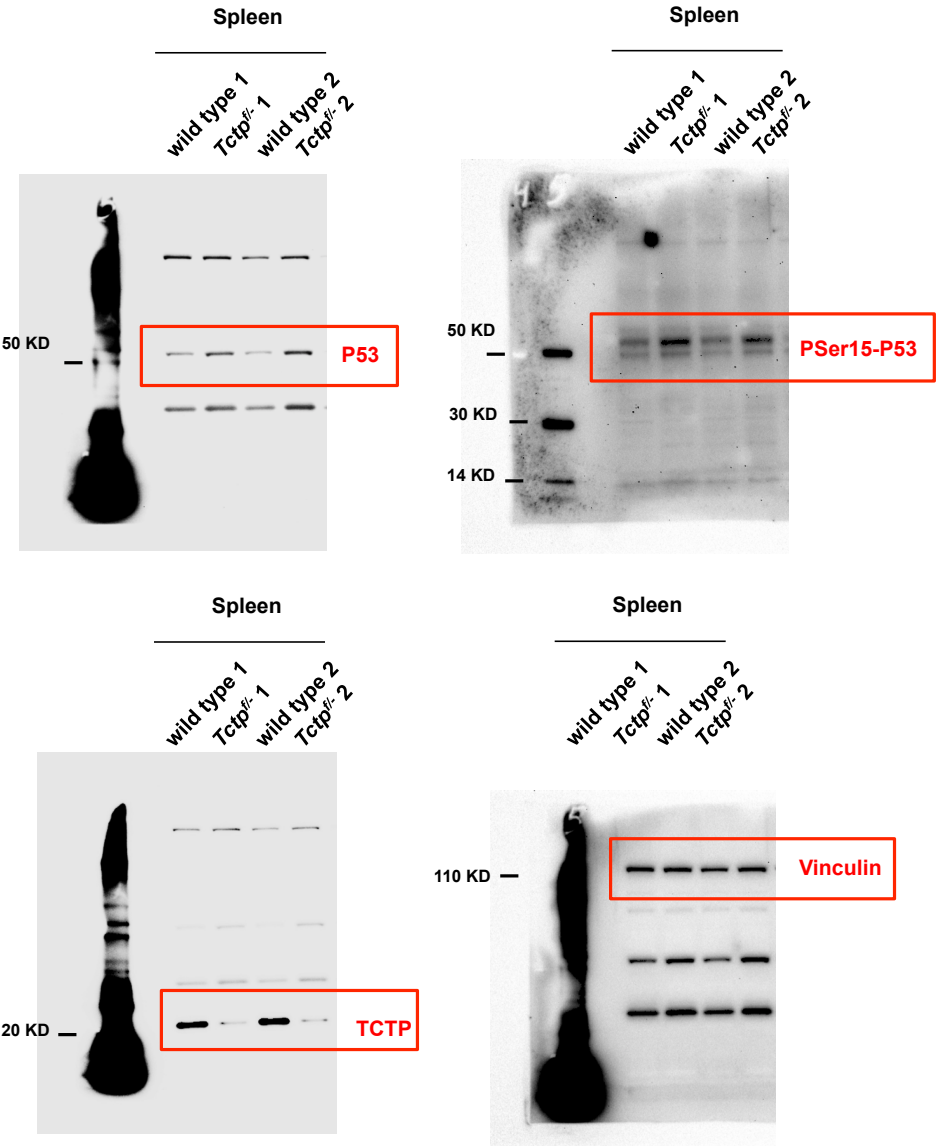

Supplement: Supplementary file 7 — Source data Fig. 1 [file 44319_2024_108_MOESM7_ESM.zip › Source Data Figure 1/Source Data Fig 1C.pdf]

Fig 1B

Western blots presented in the manuscript:

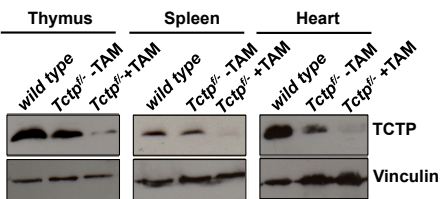

Original uncropped Western blots:

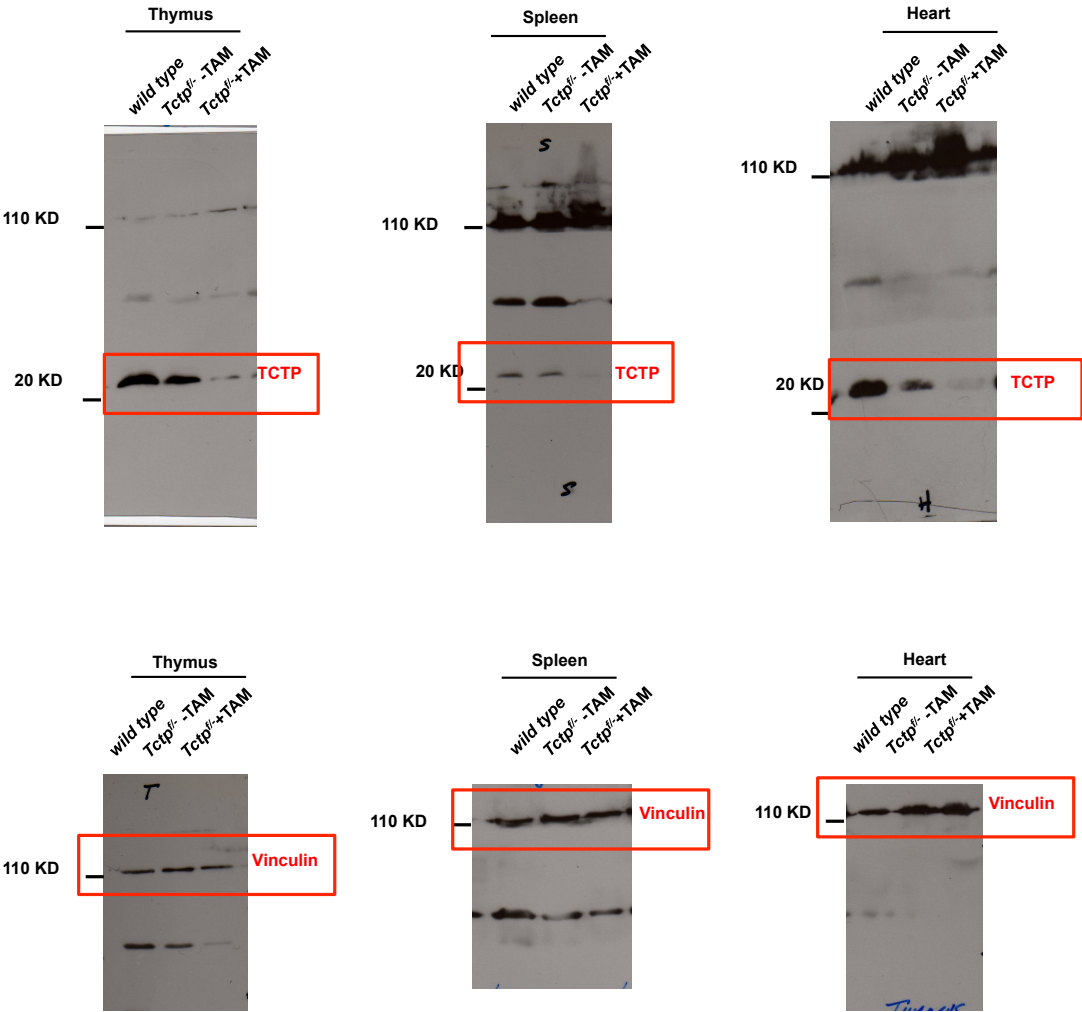

Supplement: Supplementary file 7 — Source data Fig. 1 [file 44319_2024_108_MOESM7_ESM.zip › Source Data Figure 1/Source Data Fig 1B.pdf]

Fig 2B

Western blots presented in the manuscript:

Original uncropped Western blots:

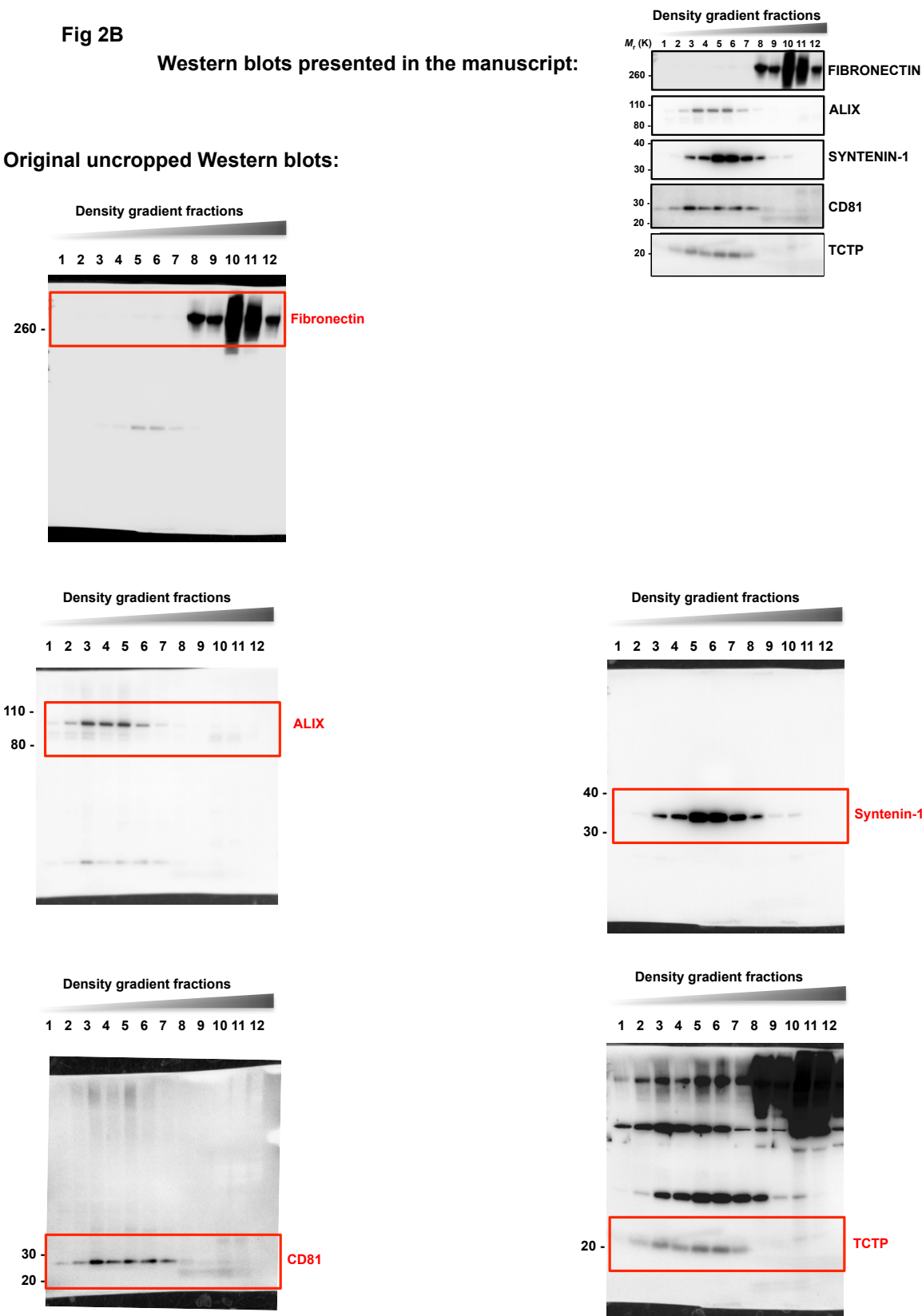

Supplement: Supplementary file 8 — Source data Fig. 2 [file 44319_2024_108_MOESM8_ESM.zip › Source Data Figure 2/Source Data Fig 2B.pdf]

**Fig 2A** Western blots presented in the manuscript:

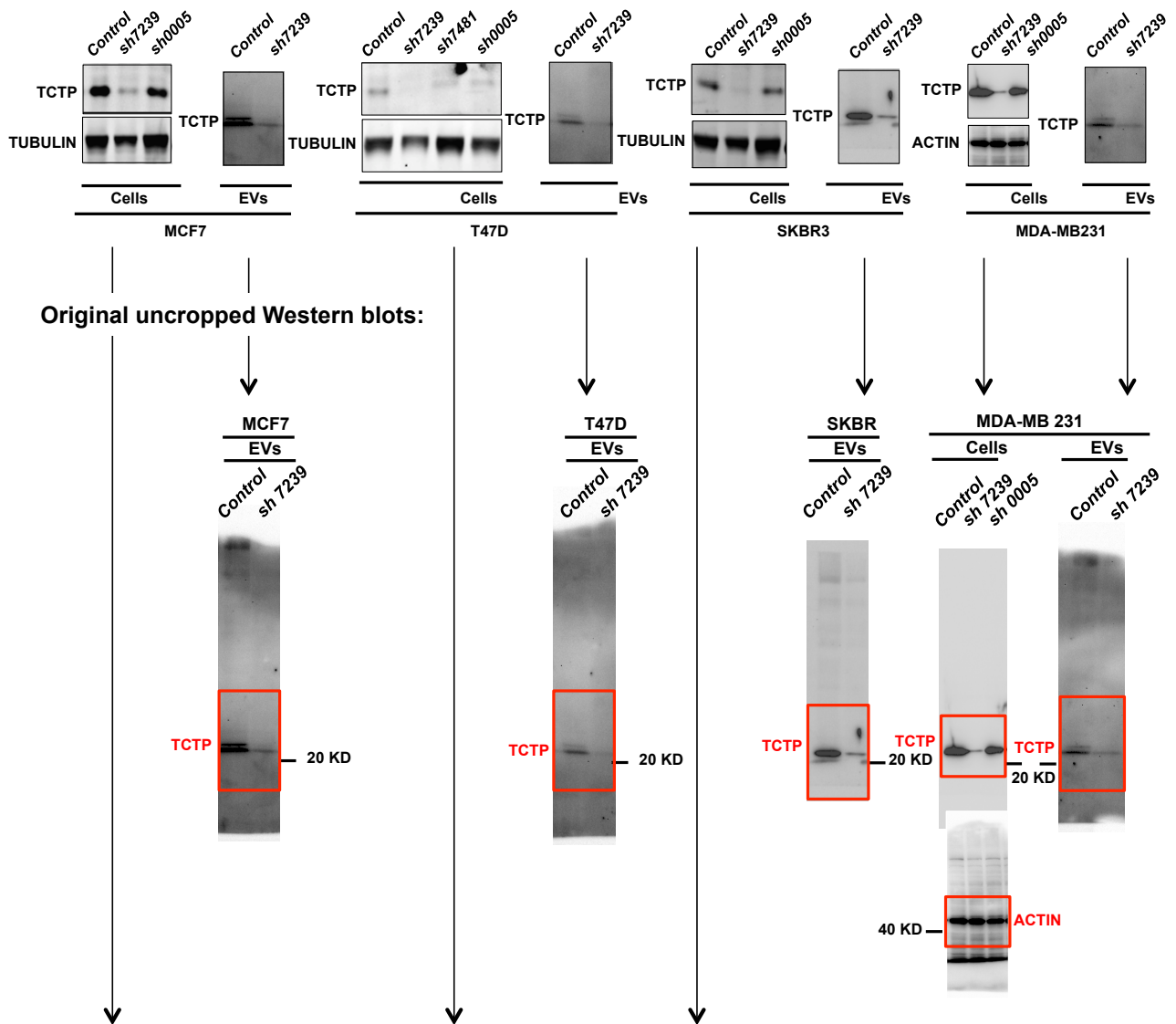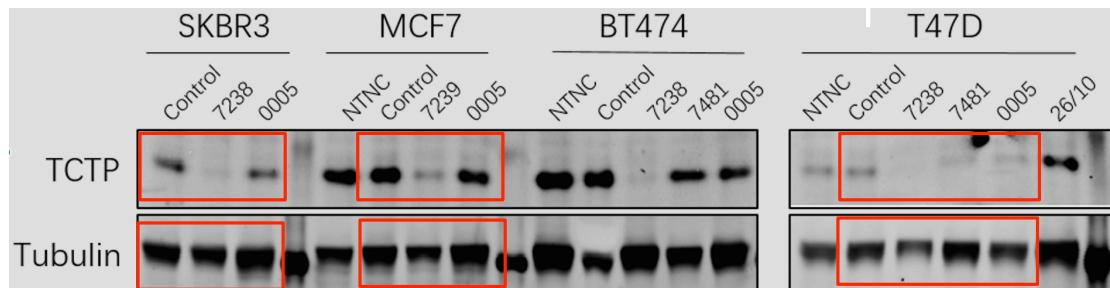

Supplement: Supplementary file 8 — Source data Fig. 2 [file 44319_2024_108_MOESM8_ESM.zip › Source Data Figure 2/Source Data Fig 2A.pdf]

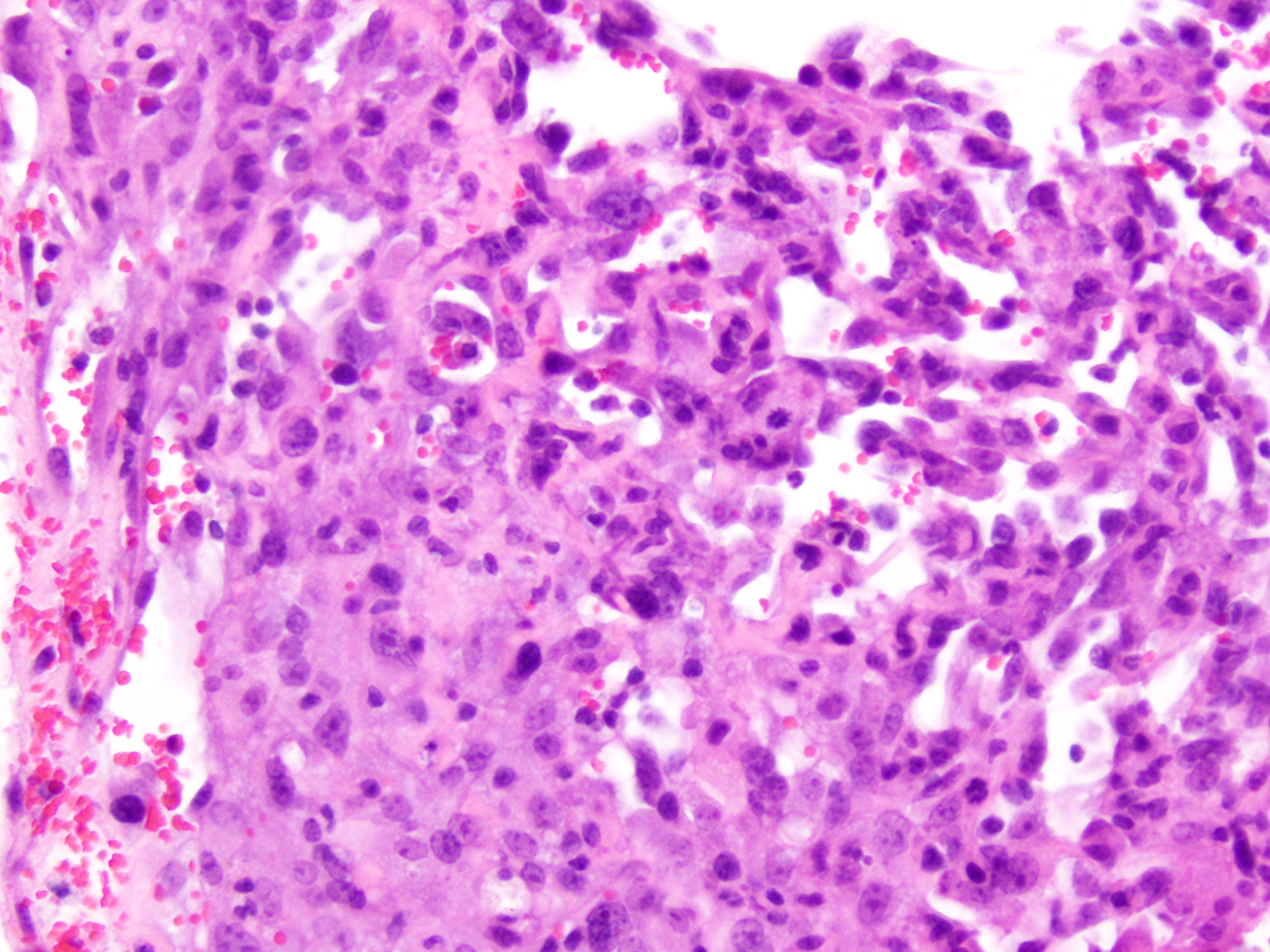

Supplement: Supplementary file 9 — Source data Fig. 3 [file 44319_2024_108_MOESM9_ESM.zip › Source Data Figure 3 /Source Data Fig 3H .TIF]

**Fig 3A Western blots presented in the manuscript:**

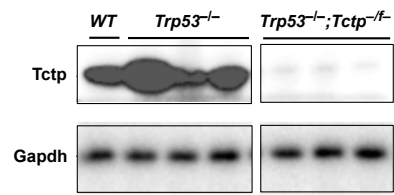

**Original uncropped Western blots:**

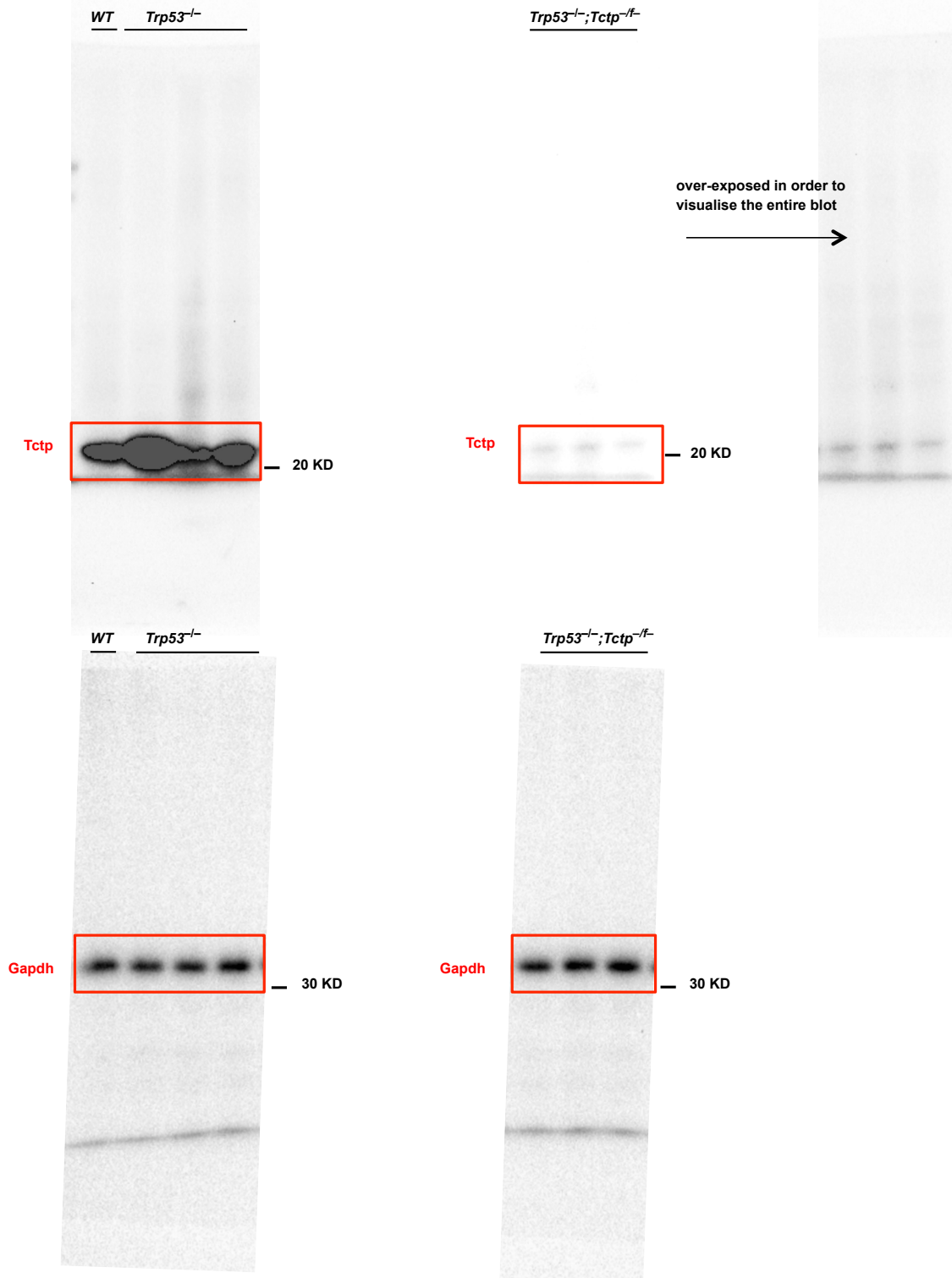

Supplement: Supplementary file 9 — Source data Fig. 3 [file 44319_2024_108_MOESM9_ESM.zip › Source Data Figure 3 /Source Data Fig 3A.pdf]

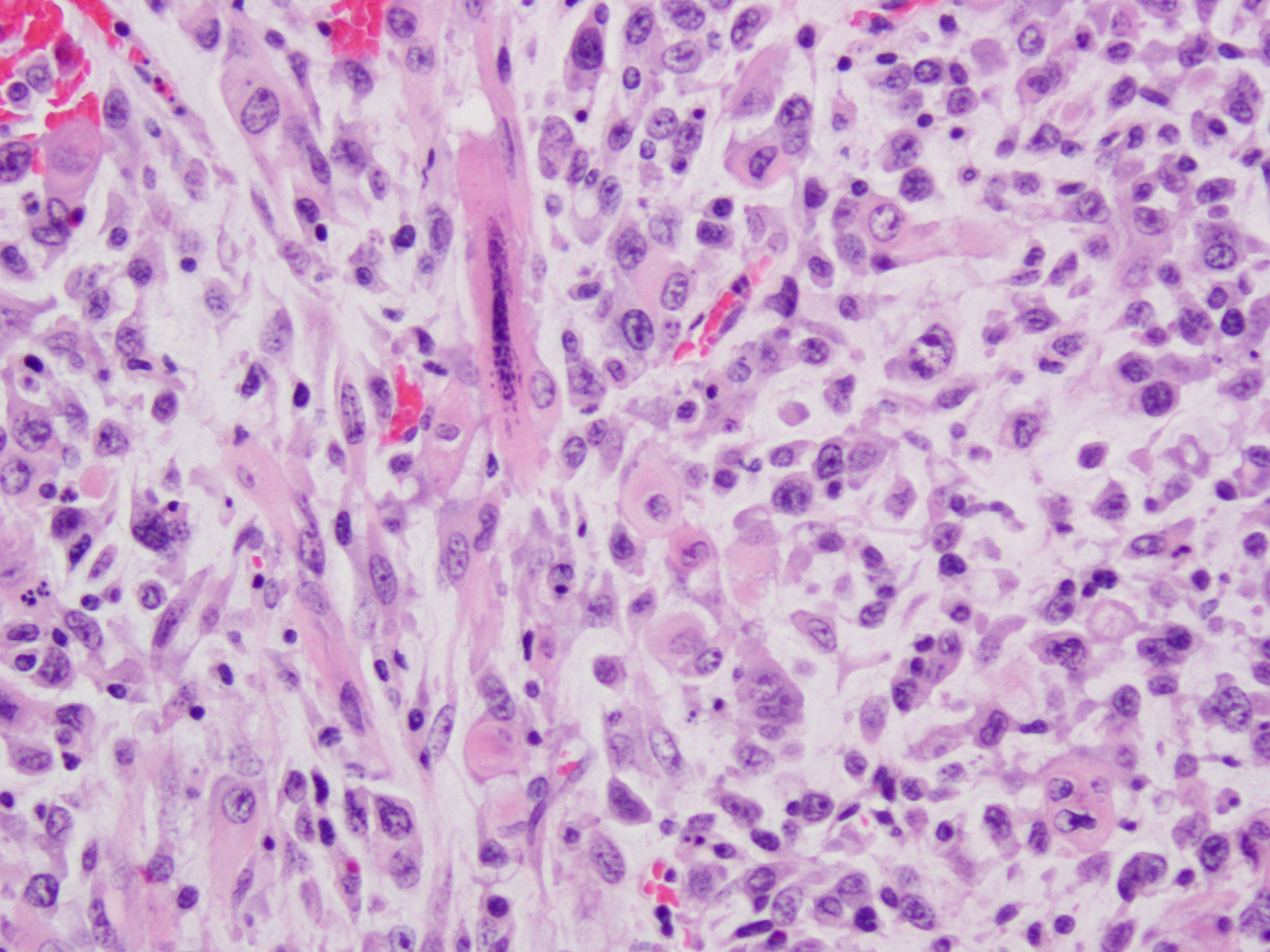

Supplement: Supplementary file 9 — Source data Fig. 3 [file 44319_2024_108_MOESM9_ESM.zip › Source Data Figure 3 /Source Data Fig 3I .TIF]

**Fig 3C**

**Western blots presented in the manuscript:**

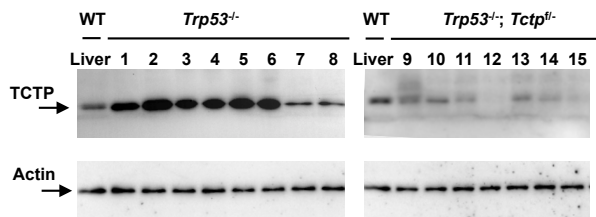

**Original uncropped Western blots:**

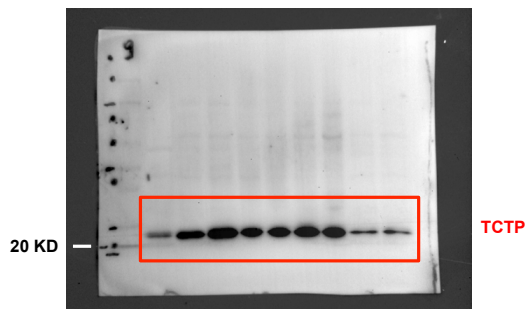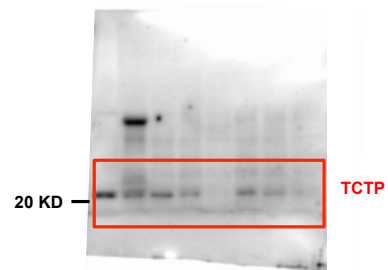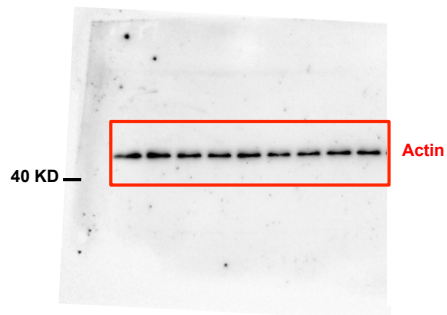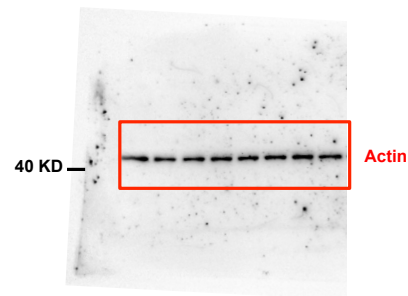

Supplement: Supplementary file 9 — Source data Fig. 3 [file 44319_2024_108_MOESM9_ESM.zip › Source Data Figure 3 /Source Data Fig 3C.pdf]

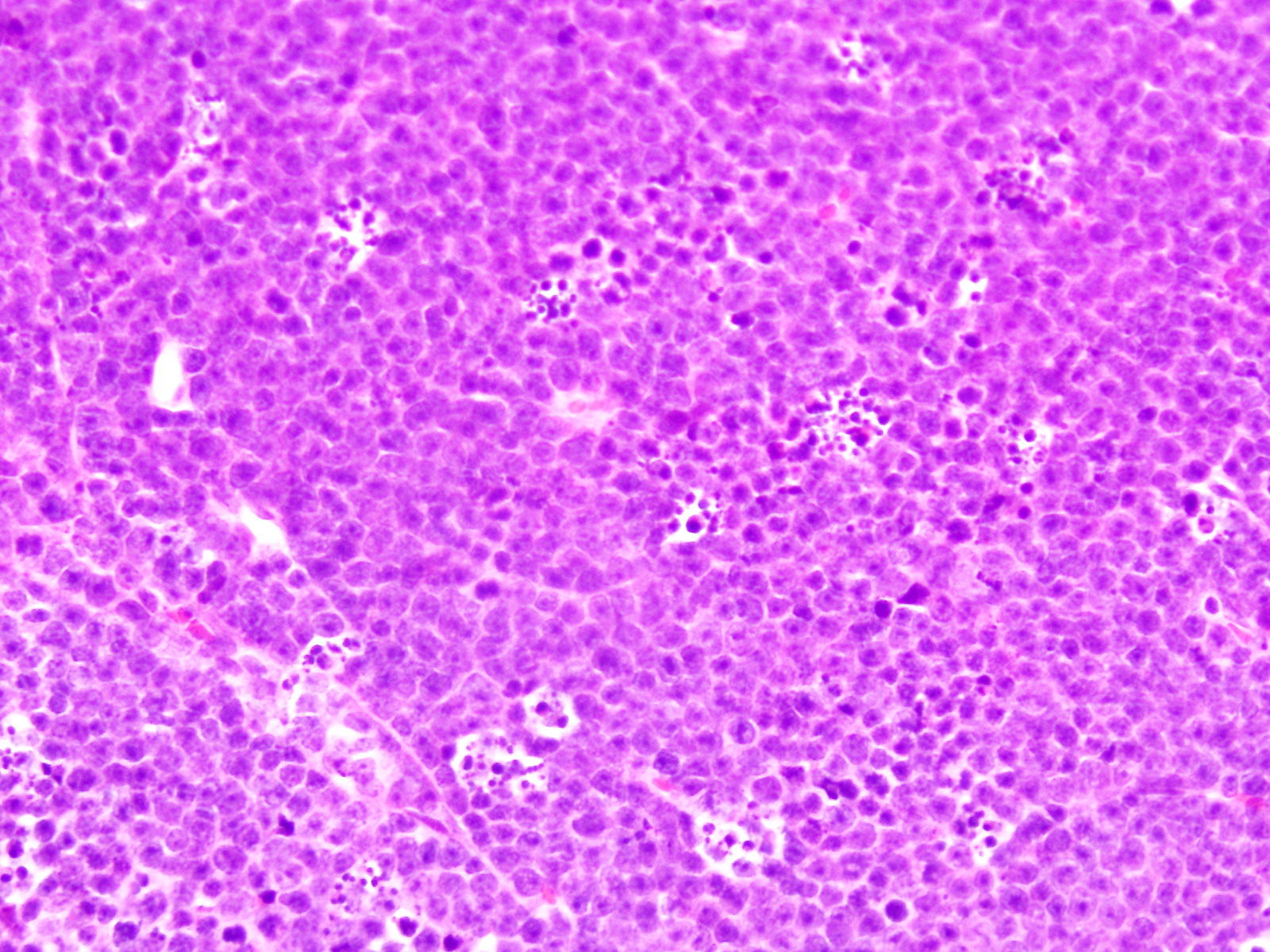

Supplement: Supplementary file 9 — Source data Fig. 3 [file 44319_2024_108_MOESM9_ESM.zip › Source Data Figure 3 /Source Data Fig 3G .TIF]

Fig 4A

Western blots presented in the manuscript:

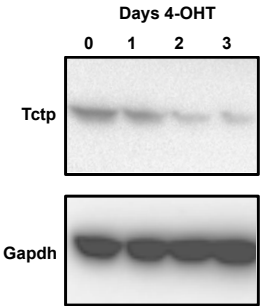

Original uncropped Western blots:

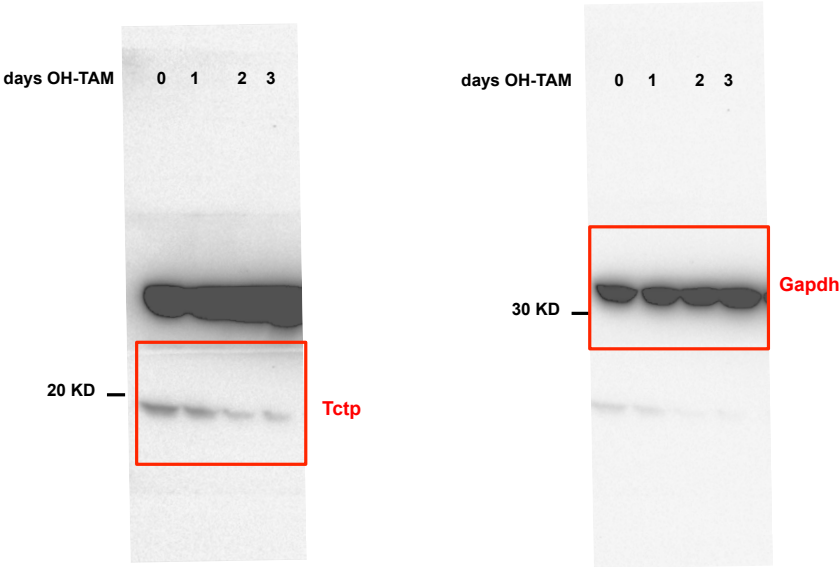

Supplement: Supplementary file 10 — Source data Fig. 4 [file 44319_2024_108_MOESM10_ESM.zip › Source Data Figure 4 /Source Data Fig 4A.pdf]

Fig 4B

Western blots presented in the manuscript:

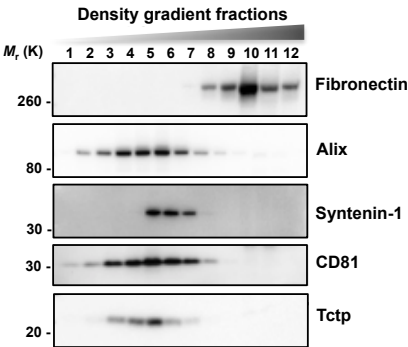

Original uncropped Western blots:

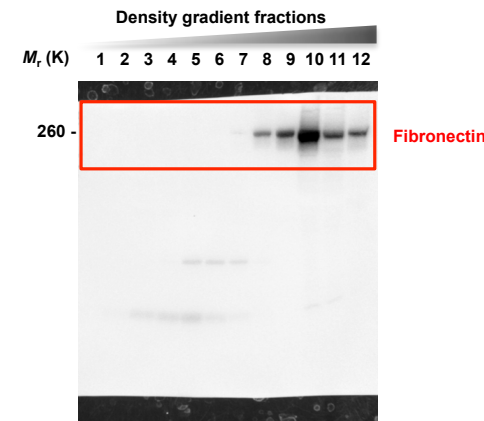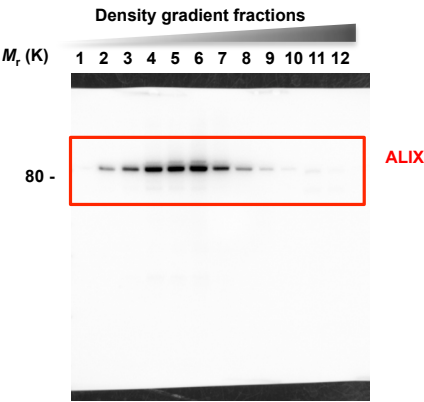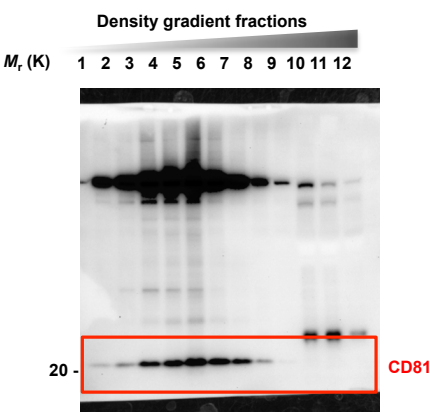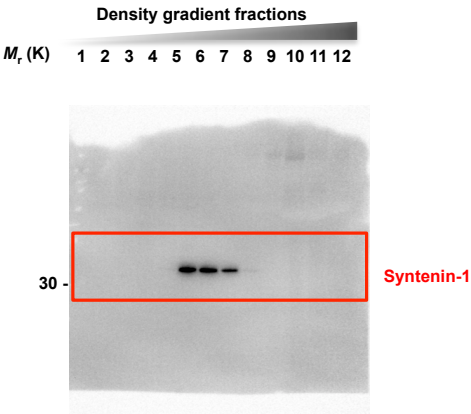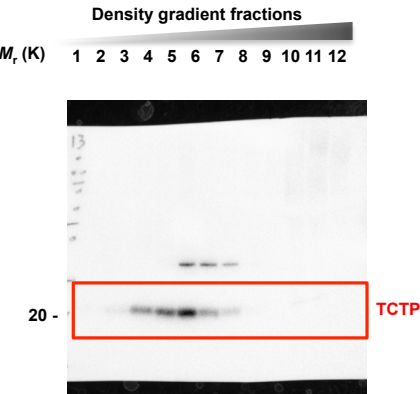

Supplement: Supplementary file 10 — Source data Fig. 4 [file 44319_2024_108_MOESM10_ESM.zip › Source Data Figure 4 /Source Data Fig 4B.pdf]

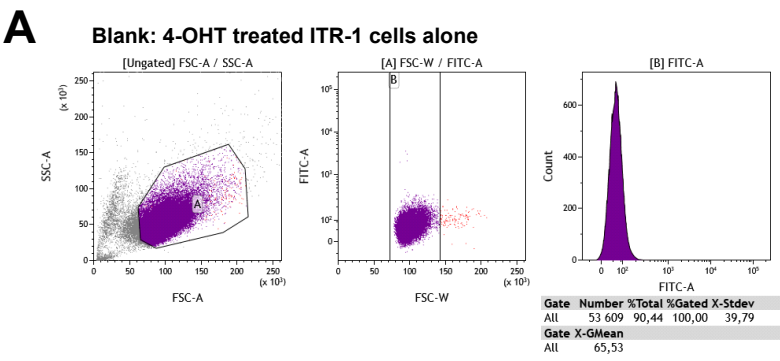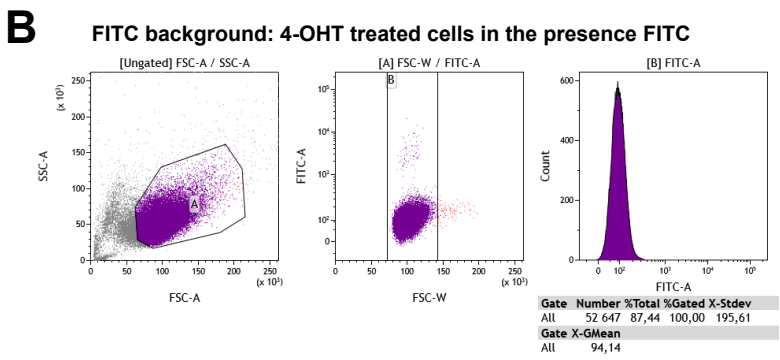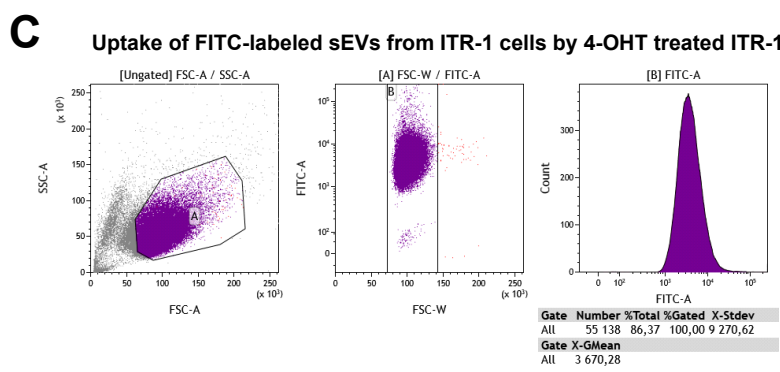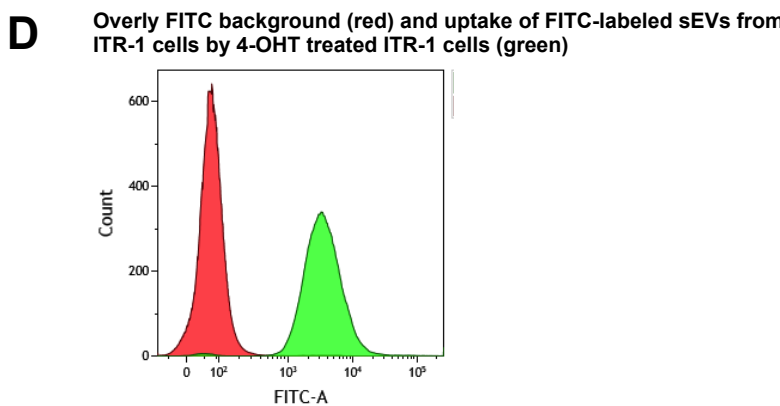

Figure 4G

Supplement: Supplementary file 10 — Source data Fig. 4 [file 44319_2024_108_MOESM10_ESM.zip › Source Data Figure 4 /Source Data Fig 4G.pdf]

**Fig 4C Inlay**

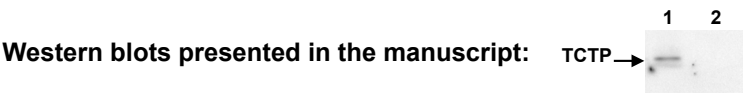

**Original uncropped Western blots:**

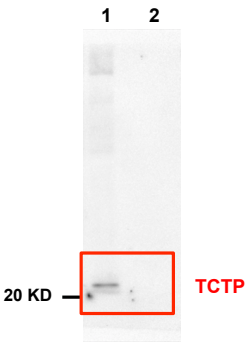

Supplement: Supplementary file 10 — Source data Fig. 4 [file 44319_2024_108_MOESM10_ESM.zip › Source Data Figure 4 /Source Data Fig 4C Inlay.pdf]

Fig 5A

Western blots presented in the manuscript:

Original uncropped Western blots:

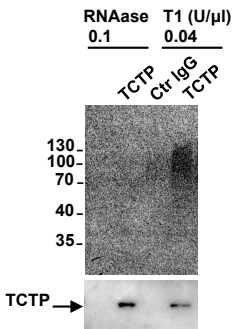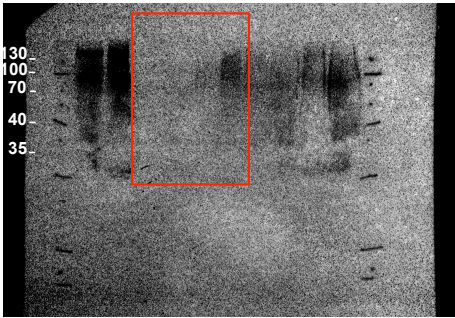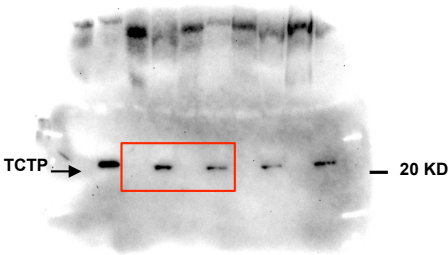

Supplement: Supplementary file 11 — Source data Fig. 5 [file 44319_2024_108_MOESM11_ESM.zip › Source Data Figure 5/Source Data Fig 5A.pdf]

## Appendix S6

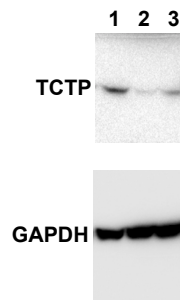

1. ITR-1
2. ITR-1 + OHT
3. ITR-1 + OHT + sEVs from ITR-1

Original uncropped Western blots:

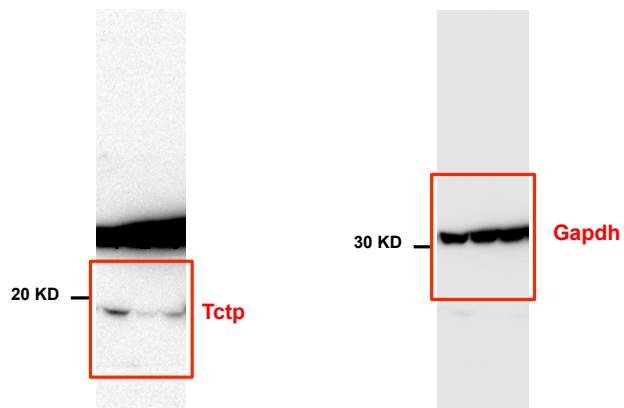

Supplement: Supplementary file 12 — EV and Appendix Figure Source Data [file 44319_2024_108_MOESM12_ESM.zip › EMBOR-2023-58195V3-EV_and_Appendix_Figure_Source_Data-sd/Source Data Appendix/Source Data Appendix S6 .pdf]

## Appendix S1E

Gel presented in Appendix:

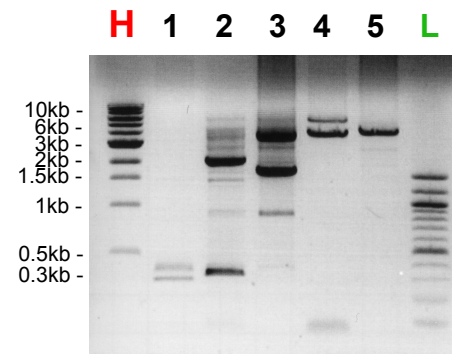

Original uncropped gel:

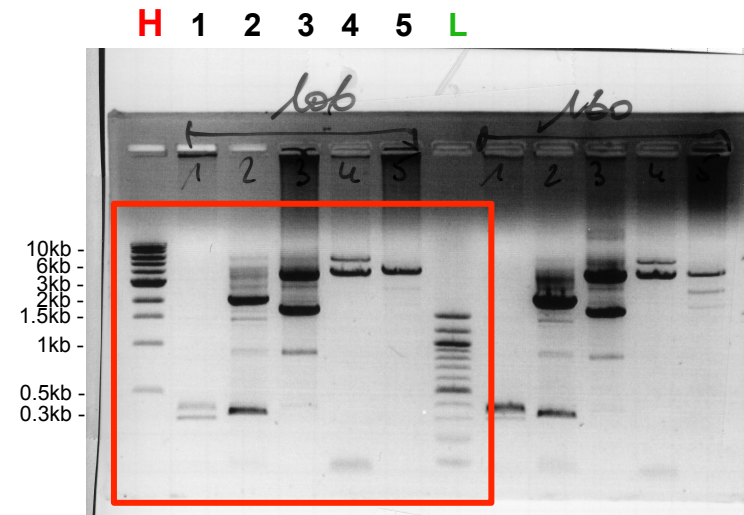

Supplement: Supplementary file 12 — EV and Appendix Figure Source Data [file 44319_2024_108_MOESM12_ESM.zip › EMBOR-2023-58195V3-EV_and_Appendix_Figure_Source_Data-sd/Source Data Appendix/Source Data Appendix S1E.pdf]

## Appendix S1I

mouse A

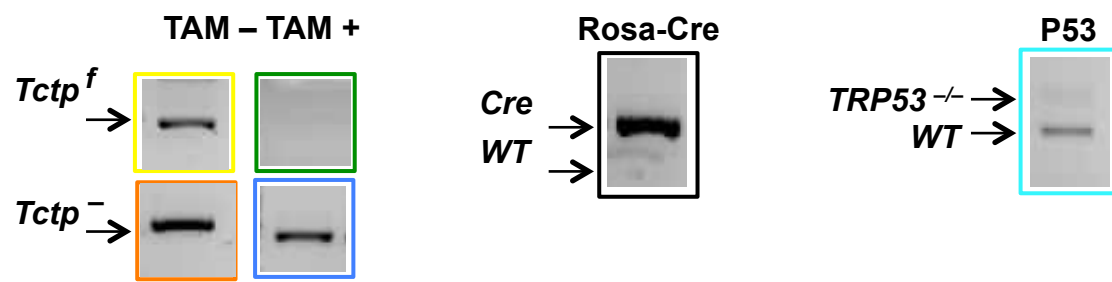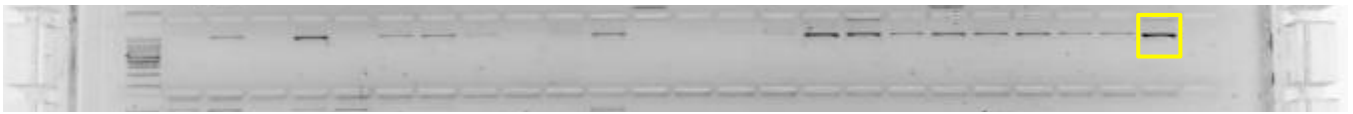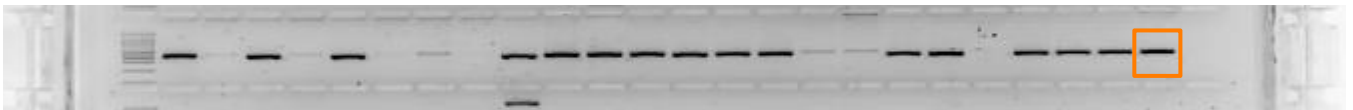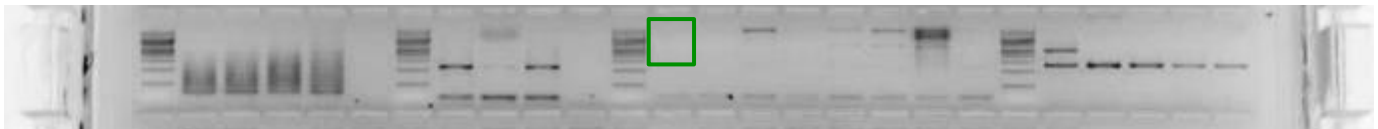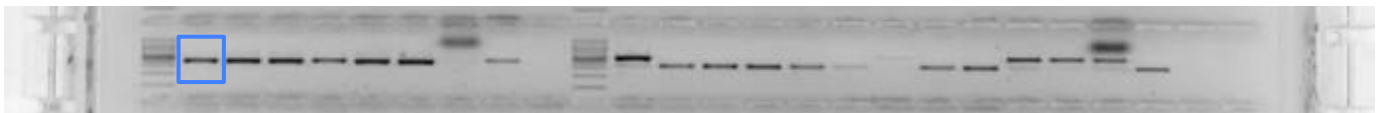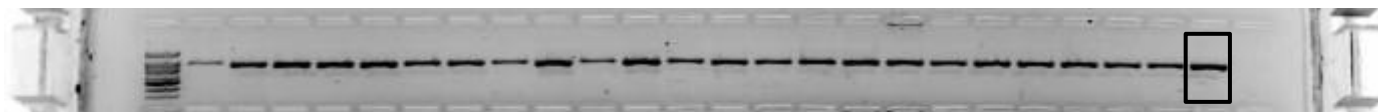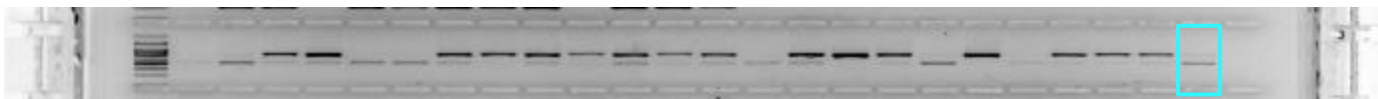

mouse B

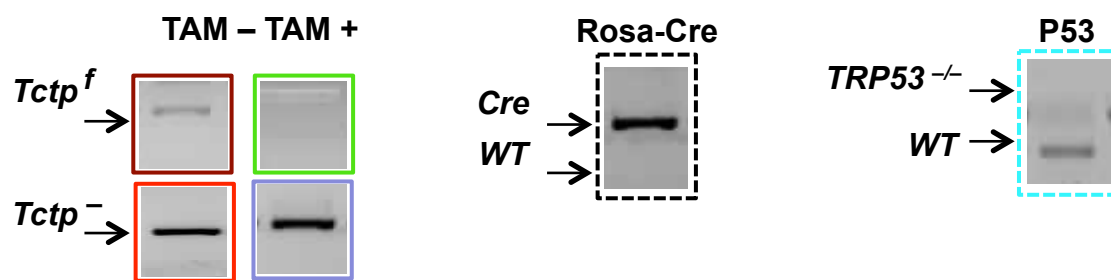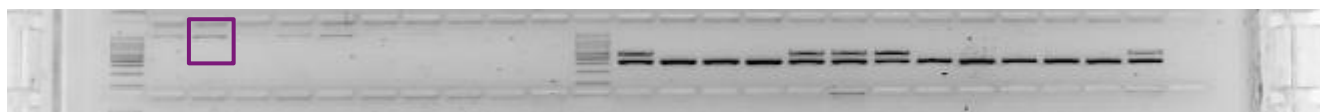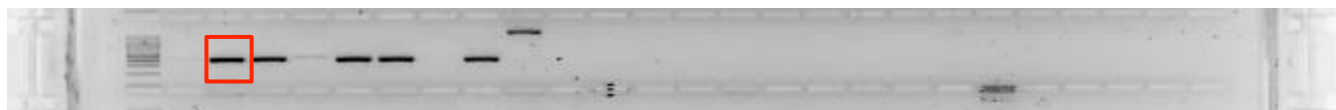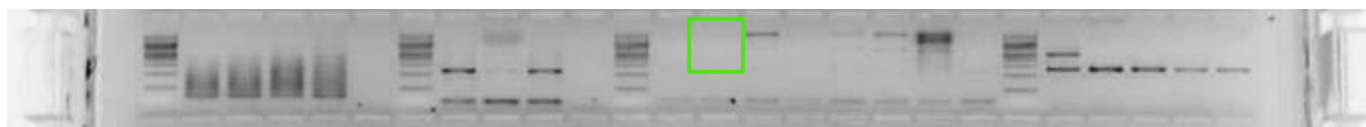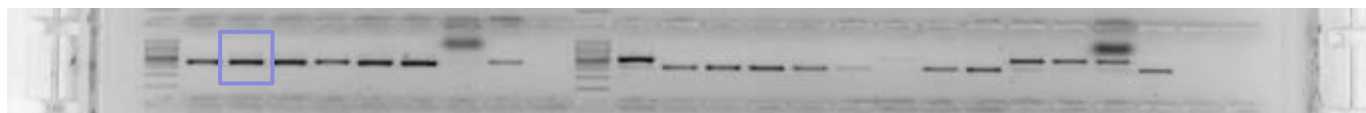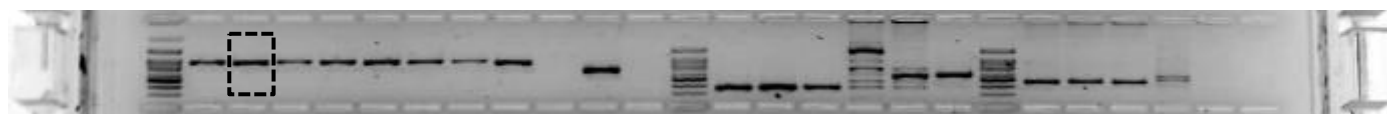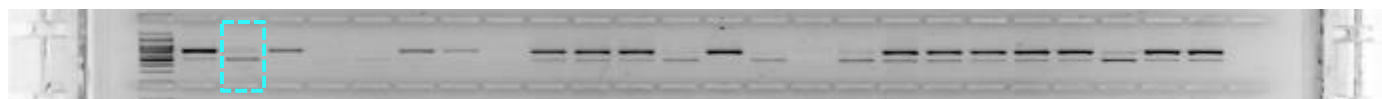

mouse C

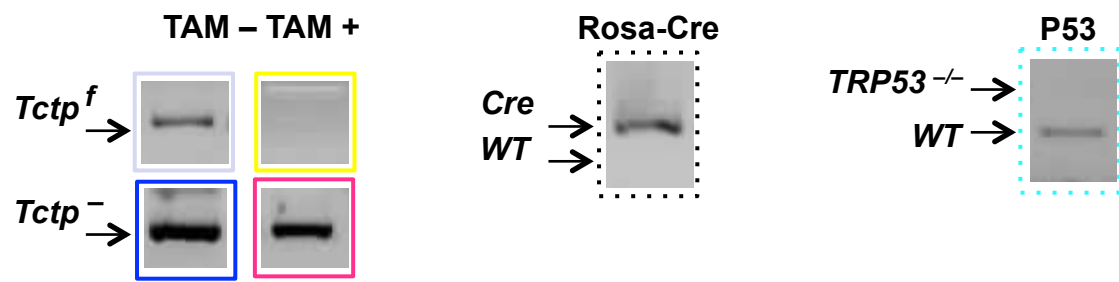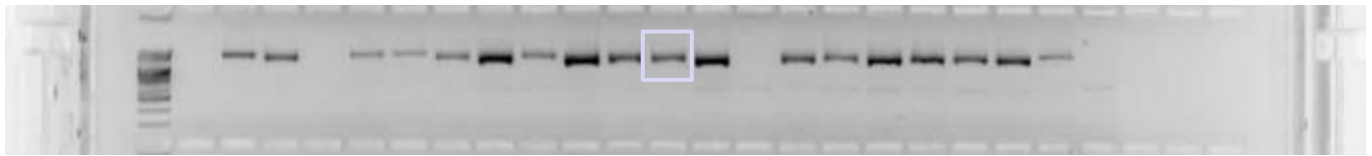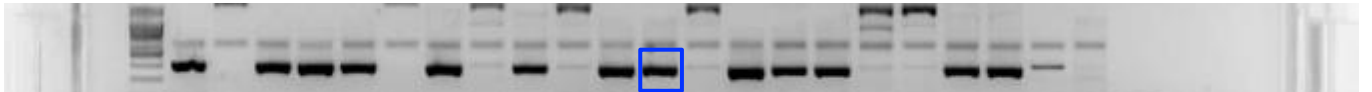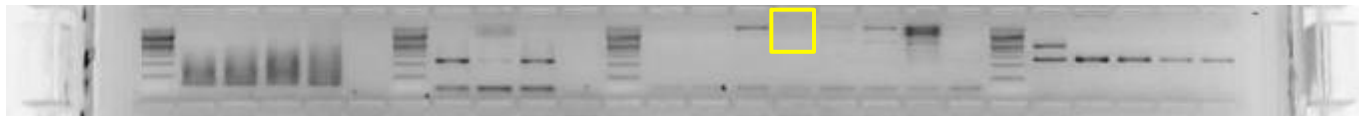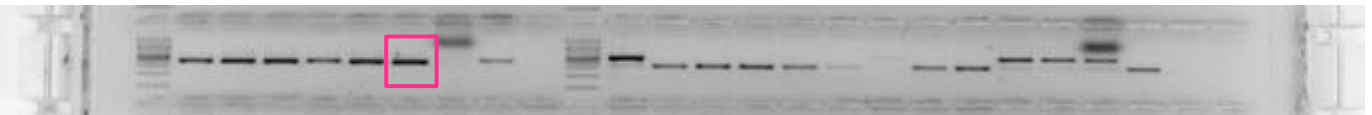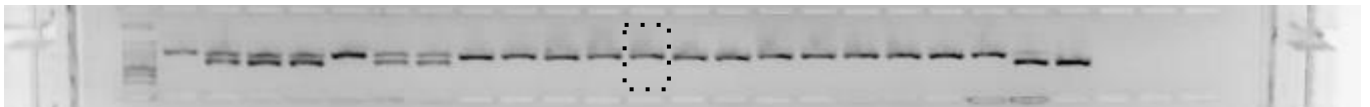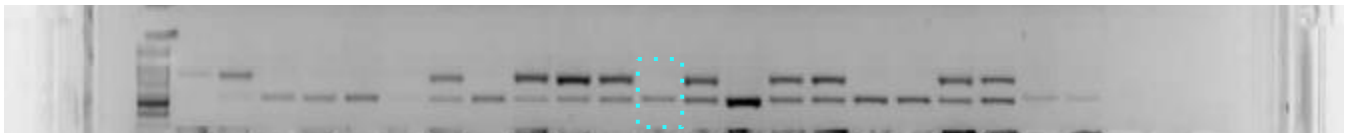

Supplement: Supplementary file 12 — EV and Appendix Figure Source Data [file 44319_2024_108_MOESM12_ESM.zip › EMBOR-2023-58195V3-EV_and_Appendix_Figure_Source_Data-sd/Source Data Appendix/Source Data Appendix S1I.pdf]
