# Supplementary material for: TCTP regulates genotoxic stress and tumorigenicity via intercellular vesicular signaling
Source: EMBO Rep. 2024 Mar 28;25(4):20. doi: 10.1038/s44319-024-00108-7 (PMC11014985; doi:10.1038/s44319-024-00108-7)
Supplement: Supplementary file 8 — Source data Fig. 2 [file 44319_2024_108_MOESM8_ESM.zip › Source Data Figure 2/Source Data Fig 2G.pdf]

A

Blank: MCF7 sh*7239* cells alone

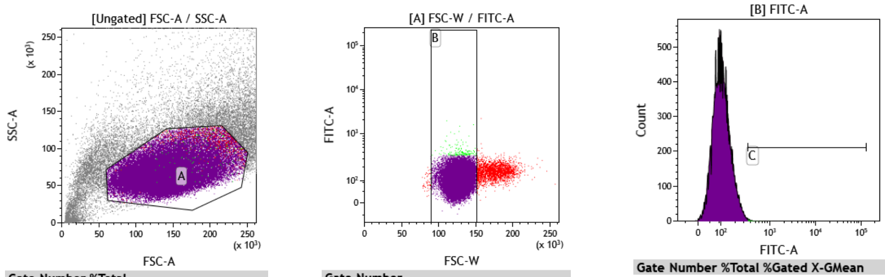

Gate Number	%Total
All	72,884 100.00
A	50,585 69.40

Gate Number	
All	50,585
B	48,120

Gate Number	%Total	%Gated	X-GMean
All 48,120	66.02	100.00	103.87
C 154	0.21	0.32	438.12

B

FITC background: MCF7 sh*7239* cells in the presence FITC

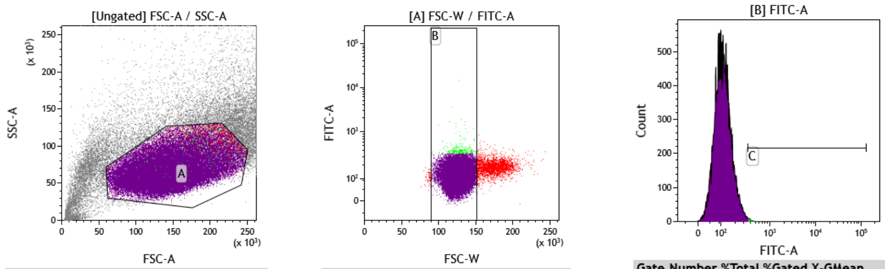

Gate Number	%Total
All	77,263 100.00
A	52,760 68.29

Gate Number	
All	52,760
B	50,232

Gate Number	%Total	%Gated	X-GMean
All 50,232	65.01	100.00	111.65
C 251	0.32	0.50	439.25

C

Uptake of FITC-labeled sEVs derived from MCF7 cells by MCF7 sh*7239* cells

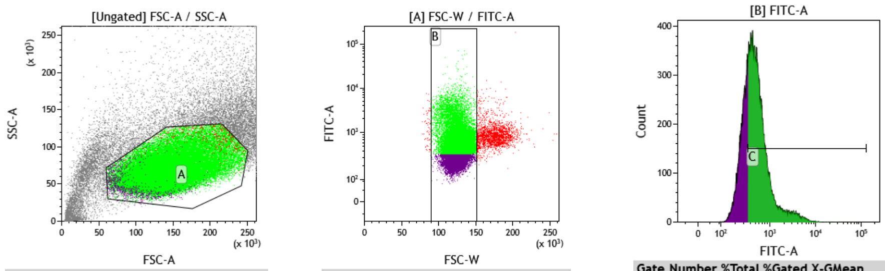

Gate Number	%Total
All	72,316 100.00
A	50,418 69.72

Gate Number	
All	50,418
B	48,235

Gate Number	%Total	%Gated	X-GMean
All 48,235	66.70	100.00	499.72
C 34,847	48.19	72.24	637.75

D

Overlay blank (red) and uptake of FITC-labeled sEVs
derived from MCF7 cells by MCF7 sh*7239* cells (green)

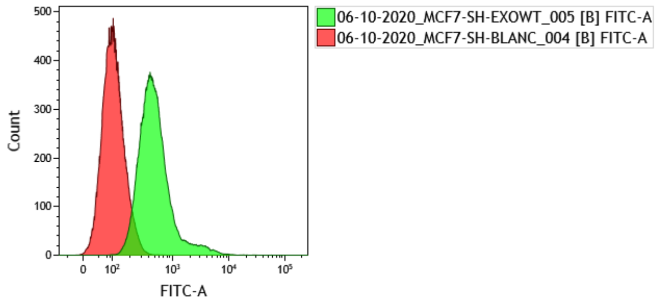

Figure 2G
