# Supplementary material for: TCTP regulates genotoxic stress and tumorigenicity via intercellular vesicular signaling
Source: EMBO Rep. 2024 Mar 28;25(4):20. doi: 10.1038/s44319-024-00108-7 (PMC11014985; doi:10.1038/s44319-024-00108-7)
Supplement: Supplementary file 8 — Source data Fig. 2 [file 44319_2024_108_MOESM8_ESM.zip › Source Data Figure 2/Source Data Fig 2C Right.pdf]

NANOSIGHT

MCF7SH39 1-200SEV 2020-01-22 15-07-45

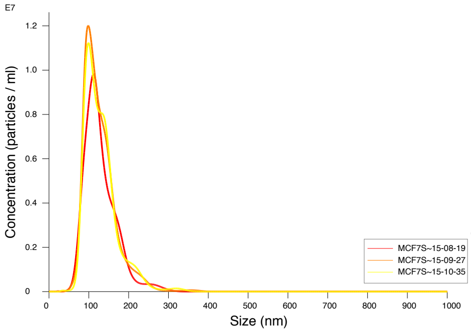

FTLA Concentration / Size graph for Experiment:
MCF7SH39 1-200SEV 2020-01-22 15-07-45

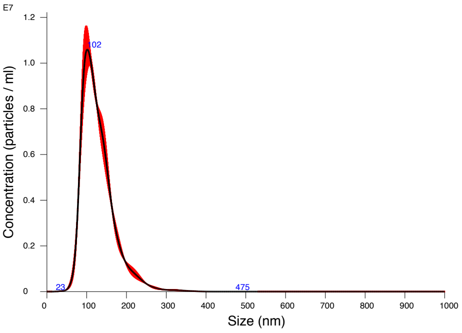

Averaged FTLA Concentration / Size for Experiment:
MCF7SH39 1-200SEV 2020-01-22 15-07-45
Error bars indicate + / -1 standard error of the mean

Included Files

MCF7SH39 1-200SEV 2020-01-22 15-08-19
MCF7SH39 1-200SEV 2020-01-22 15-09-27
MCF7SH39 1-200SEV 2020-01-22 15-10-35

Details

NTA Version:	NTA 3.3 - Sample Assistant Dev Build 3.3.203
Script Used:	SOP Standard Measurement 03-05-34PM 22J~
Time Captured:	15:07:45 22/01/2020
Operator:	MCF7SH39 1-200SEV
Pre-treatment:	
Sample Name:	test
Diluent:	
Remarks:	

Capture Settings

Camera Type:	sCMOS
Laser Type:	Blue405
Camera Level:	15
Slider Shutter:	1206
Slider Gain:	366
FPS	25.0
Number of Frames:	1498
Temperature:	18.2 ^{o}C
Viscosity:	(Water) 1.043 - 1.045 cP
Dilution factor:	Dilution not recorded
Syringe Pump Speed:	30

Analysis Settings

Detect Threshold:	4
Blur Size:	Auto
Max Jump Distance:	Auto: 13.5 - 14.0 pix

Results

Stats: Merged Data

Mean:	128.5 nm
Mode:	101.6 nm
SD:	41.1 nm
D10:	86.8 nm
D50:	120.1 nm
D90:	180.1 nm

Stats: Mean +/- Standard Error

Mean:	128.5 +/- 0.7 nm
Mode:	103.2 +/- 4.4 nm
SD:	41.0 +/- 0.5 nm
D10:	86.7 +/- 0.7 nm
D50:	120.1 +/- 0.8 nm
D90:	180.3 +/- 1.6 nm
Concentration (Upgrade):	8.15e+08 +/- 3.83e+07 particles/ml
	125.0 +/- 5.7 particles/frame
	124.6 +/- 2.4 centres/frame

Figure 2C Right Amson et al.
