# Supplementary material for: TCTP regulates genotoxic stress and tumorigenicity via intercellular vesicular signaling
Source: EMBO Rep. 2024 Mar 28;25(4):20. doi: 10.1038/s44319-024-00108-7 (PMC11014985; doi:10.1038/s44319-024-00108-7)
Supplement: Supplementary file 8 — Source data Fig. 2 [file 44319_2024_108_MOESM8_ESM.zip › Source Data Figure 2/Source Data Fig 2C Left.pdf]

NANOSIGHT

MCF7CTR1-20SEV 2020-01-16 17-47-41

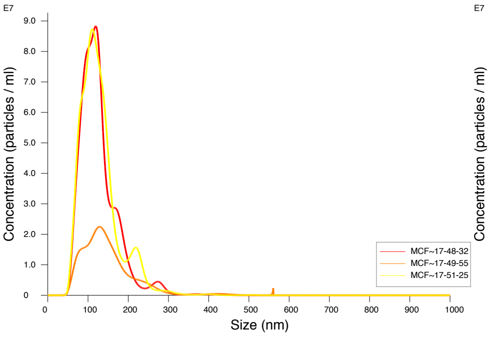

FTLA Concentration / Size graph for Experiment:
MCF7CTR1-20SEV 2020-01-16 17-47-41

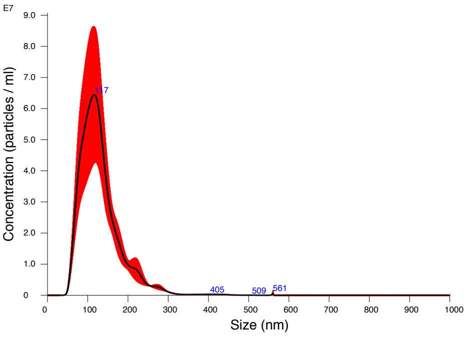

Averaged FTLA Concentration / Size for Experiment:
MCF7CTR1-20SEV 2020-01-16 17-47-41
Error bars indicate + / -1 standard error of the mean

Included Files

MCF7CTR1-100SEV 2020-01-16 17-48-32
MCF7CTR1-100SEV 2020-01-16 17-49-55
MCF7CTR1-100SEV 2020-01-16 17-51-25

Details

NTA Version:	NTA 3.3 - Sample Assistant Dev Build 3.3.203
Script Used:	SOP Standard Measurement 05-33-01PM 16J~
Time Captured:	17:47:41 16/01/2020
Operator:	MCF7CTR1-5SEV
Pre-treatment:	
Sample Name:	test
Diluent:	
Remarks:	

Capture Settings

Camera Type:	Unknown
Laser Type:	Unknown
Camera Level:	0 - 12
Slider Shutter:	0 - 1200
Slider Gain:	0 - 146
FPS	25.0 - 30.0
Number of Frames:	1498
Temperature:	21.4 - 22.0 ºC
Viscosity:	(Water) 0.953 - 0.966 cP
Dilution factor:	Dilution not recorded

Analysis Settings

Detect Threshold:	4
Blur Size:	Auto
Max Jump Distance:	Auto: 11.5 - 12.7 pix

Results

Stats: Merged Data

Mean:	129.9 nm
Mode:	116.3 nm
SD:	51.0 nm
D10:	78.5 nm
D50:	120.0 nm
D90:	193.8 nm

Stats: Mean +/- Standard Error

Mean:	133.8 +/- 6.9 nm
Mode:	119.5 +/- 5.2 nm
SD:	53.5 +/- 6.3 nm
D10:	78.7 +/- 0.4 nm
D50:	123.9 +/- 5.8 nm
D90:	202.1 +/- 13.9 nm
Concentration:	5.90e+09 +/- 1.65e+09 particles/ml
	357.8 +/- 25.8 particles/frame
	315.0 +/- 1.9 centres/frame

Figure 2C Left Amson et al.
