# Supplementary material for: TCTP regulates genotoxic stress and tumorigenicity via intercellular vesicular signaling
Source: EMBO Rep. 2024 Mar 28;25(4):20. doi: 10.1038/s44319-024-00108-7 (PMC11014985; doi:10.1038/s44319-024-00108-7)
Supplement: Supplementary file 10 — Source data Fig. 4 [file 44319_2024_108_MOESM10_ESM.zip › Source Data Figure 4 /Source Data Fig 4C Right.pdf]

NANOSIGHT

ITR+TAMBC_LD_1-5_040719 2019-07-04 14-50-31

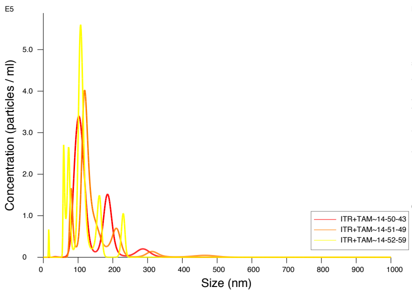

FTLA Concentration / Size graph for Experiment:
ITR+TAMBC_LD_1-5_040719 2019-07-04 14-50-31

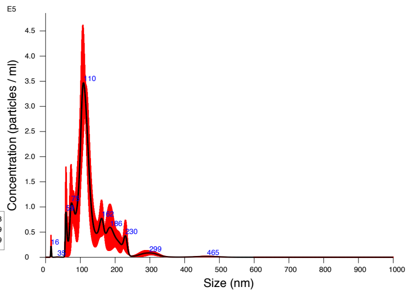

Averaged FTLA Concentration / Size for Experiment:
ITR+TAMBC_LD_1-5_040719 2019-07-04 14-50-31
Error bars indicate + / -1 standard error of the mean

Included Files

ITR+TAMBC_LD_1-5_040719 2019-07-04 14-50-43
ITR+TAMBC_LD_1-5_040719 2019-07-04 14-51-49
ITR+TAMBC_LD_1-5_040719 2019-07-04 14-52-59

Details

NTA Version:	NTA 3.3 - Sample Assistant Dev Build 3.3.203
Script Used:	SOP Standard Measurement 02-02-41PM 04J~
Time Captured:	14:50:31 04/07/2019
Operator:	ITR+TAMBC_LD_1-5_040719
Pre-treatment:	
Sample Name:	
Diluent:	
Remarks:	

Capture Settings

Camera Type:	sCMOS
Laser Type:	Blue405
Camera Level:	15 - 16
Slider Shutter:	1206 - 1300
Slider Gain:	366 - 512
FPS	25.0
Number of Frames:	1498
Temperature:	25.2 °C
Viscosity:	(Water) 0.9 cP
Dilution factor:	Dilution not recorded
Syringe Pump Speed:	50

Analysis Settings

Detect Threshold:	4
Blur Size:	Auto
Max Jump Distance:	Auto: 14.4 - 31.9 pix

Results

Stats: Merged Data

Mean:	132.7 nm
Mode:	109.1 nm
SD:	57.4 nm
D10:	79.9 nm
D50:	115.6 nm
D90:	203.1 nm

Stats: Mean +/- Standard Error

Mean:	133.1 +/- 9.8 nm
Mode:	109.5 +/- 4.9 nm
SD:	55.0 +/- 7.4 nm
D10:	81.8 +/- 7.8 nm
D50:	115.9 +/- 5.3 nm
D90:	193.1 +/- 14.8 nm
Concentration (Upgrade):	1.81e+07 +/- 3.73e+05 particles/ml
	2.5 +/- 0.2 particles/frame
	3.6 +/- 0.3 centres/frame

Concentration measurements may require some caution due to noise
See summary file for more info

Figure 4C Right
