# Supplementary material for: TCTP regulates genotoxic stress and tumorigenicity via intercellular vesicular signaling
Source: EMBO Rep. 2024 Mar 28;25(4):20. doi: 10.1038/s44319-024-00108-7 (PMC11014985; doi:10.1038/s44319-024-00108-7)
Supplement: Supplementary file 10 — Source data Fig. 4 [file 44319_2024_108_MOESM10_ESM.zip › Source Data Figure 4 /Source Data Fig 4C Left.pdf]

NANOSIGHT

ITR-TAMBC_LD_1-5_040719 2019-07-04 14-32-50

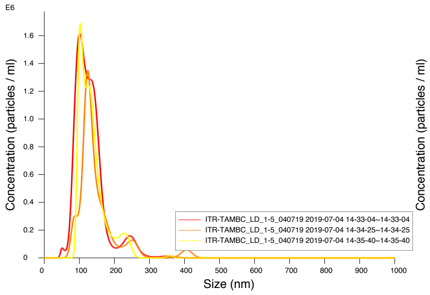

FTLA Concentration / Size graph for Experiment:
ITR-TAMBC_LD_1-5_040719 2019-07-04 14-32-50

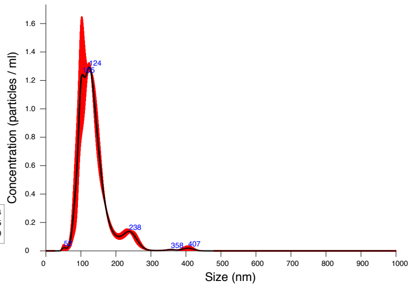

Averaged FTLA Concentration / Size for Experiment:
ITR-TAMBC_LD_1-5_040719 2019-07-04 14-32-50
Error bars indicate + / -1 standard error of the mean

Included Files

ITR-TAMBC_LD_1-5_040719 2019-07-04 14-33-04
ITR-TAMBC_LD_1-5_040719 2019-07-04 14-34-25
ITR-TAMBC_LD_1-5_040719 2019-07-04 14-35-40

Details

NTA Version:	NTA 3.3 - Sample Assistant Dev Build 3.3.203
Script Used:	SOP Standard Measurement 02-02-41PM 04J~
Time Captured:	14:32:50 04/07/2019
Operator:	ITR-TAMBC_LD_1-5_040719
Pre-treatment:	
Sample Name:	
Diluent:	
Remarks:	

Capture Settings

Camera Type:	sCMOS
Laser Type:	Blue405
Camera Level:	15
Slider Shutter:	1206
Slider Gain:	366
FPS	25.0
Number of Frames:	1498
Temperature:	24.8 - 24.8 ^{o}C
Viscosity:	(Water) 0.892 - 0.893 cP
Dilution factor:	Dilution not recorded
Syringe Pump Speed:	50

Analysis Settings

Detect Threshold:	4
Blur Size:	Auto
Max Jump Distance:	Auto: 13.7 - 17.8 pix

Results

Stats: Merged Data

Mean:	137.0 nm
Mode:	123.7 nm
SD:	48.6 nm
D10:	93.8 nm
D50:	126.5 nm
D90:	194.3 nm

Stats: Mean +/- Standard Error

Mean:	138.5 +/- 5.8 nm
Mode:	109.3 +/- 7.3 nm
SD:	48.3 +/- 6.7 nm
D10:	95.5 +/- 4.0 nm
D50:	126.6 +/- 3.0 nm
D90:	197.6 +/- 14.6 nm
Concentration (Upgrade):	9.91e+07 +/- 1.38e+07 particles/ml
	13.4 +/- 1.7 particles/frame
	14.5 +/- 1.8 centres/frame

Figure 4C Left
