# Supplementary material for: TCTP regulates genotoxic stress and tumorigenicity via intercellular vesicular signaling
Source: EMBO Rep. 2024 Mar 28;25(4):20. doi: 10.1038/s44319-024-00108-7 (PMC11014985; doi:10.1038/s44319-024-00108-7)

Fig 5L

Western blots presented in the manuscript:
Non-denaturating gel

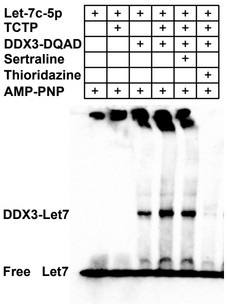

Original uncropped Western blots:
Non-denaturating gel

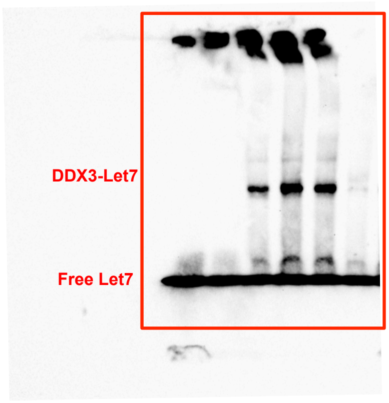

over-exposed in order to visualise the entire blot

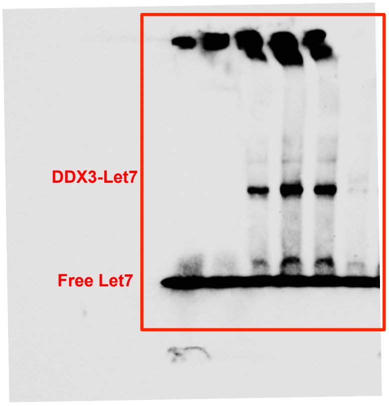

Supplement: Supplementary file 11 — Source data Fig. 5 [file 44319_2024_108_MOESM11_ESM.zip › Source Data Figure 5/Source Data Fig 5L.pdf]
