# Supplementary material for: TCTP regulates genotoxic stress and tumorigenicity via intercellular vesicular signaling
Source: EMBO Rep. 2024 Mar 28;25(4):20. doi: 10.1038/s44319-024-00108-7 (PMC11014985; doi:10.1038/s44319-024-00108-7)
Supplement: Supplementary file 14 — Expanded View Figures [file 44319_2024_108_MOESM14_ESM.pdf]

Expanded View Figures

Figure EV1. Effect of sEVs on apoptosis measured by TUNEL.

(**A**) TUNEL assay on reporter cells alone (*WT* thymocytes). (**B**) Reporter thymocytes co-cultured with sEVs derived from γ-irradiated *WT* thymocytes. (**C**) Reporter thymocytes co-cultured with sEVs derived from γ-irradiated $Tctp^{-/f-}$ thymocytes. (**D**) Relative percentage of TUNEL positive cells. Mean ± SEM (independent biological replicates $n = 4$ conditions) (ANOVA ***$P < 0.001$). Data information: (**A–D**) Scale bars: 400 μm. Source data are available online for this figure.

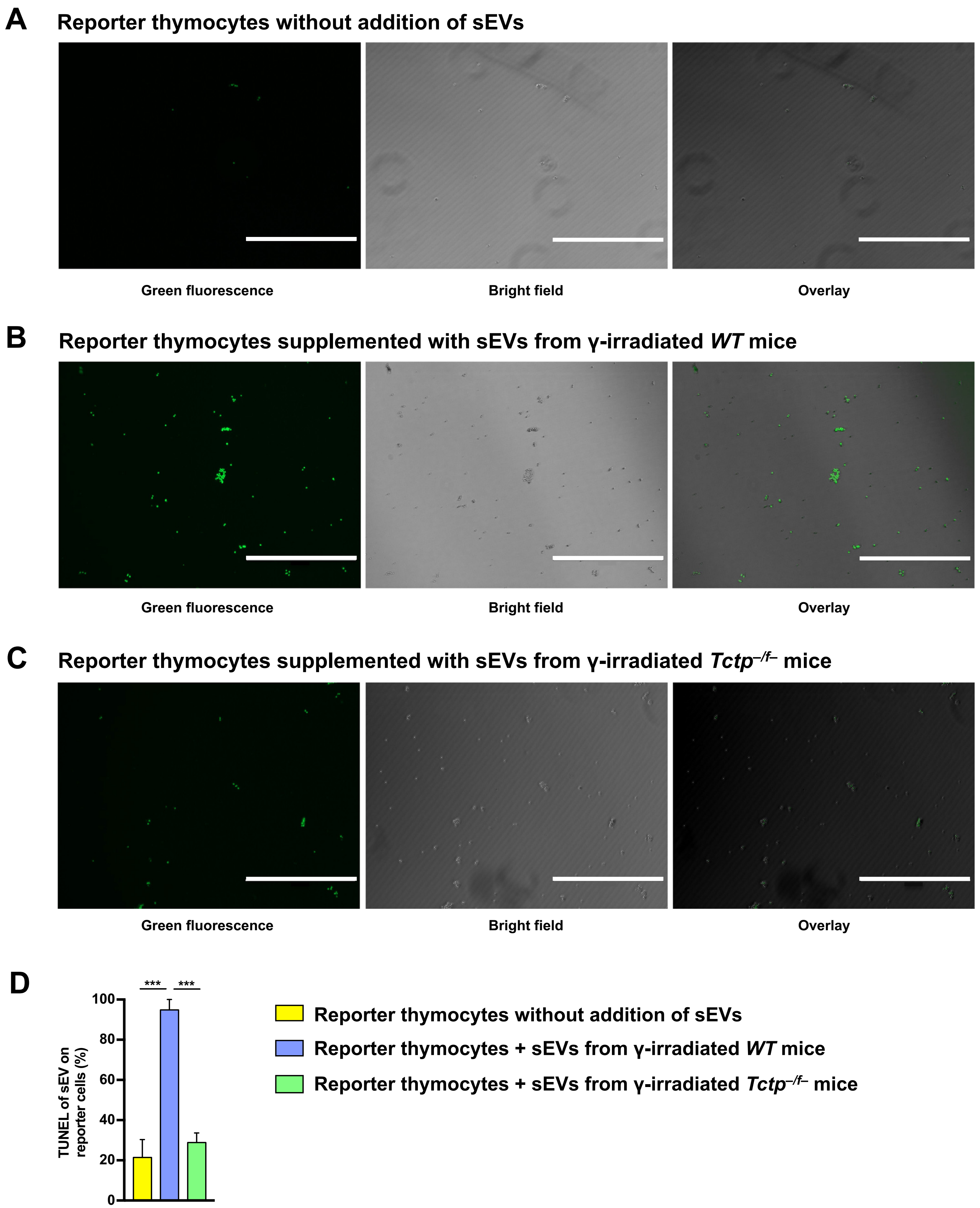
A
Reporter thymocytes without addition of sEVs
Green fluorescence
Bright field
Overlay
B
Reporter thymocytes supplemented with sEVs from γ-irradiated WT mice
Green fluorescence
Bright field
Overlay
C
Reporter thymocytes supplemented with sEVs from γ-irradiated Tctp−/f− mice
Green fluorescence
Bright field
Overlay
D
TUNEL of sEV on reporter cells (%)
0
20
40
60
80
100
***
***
Reporter thymocytes without addition of sEVs
Reporter thymocytes + sEVs from γ-irradiated WT mice
Reporter thymocytes + sEVs from γ-irradiated Tctp−/f− mice

© The Author(s)

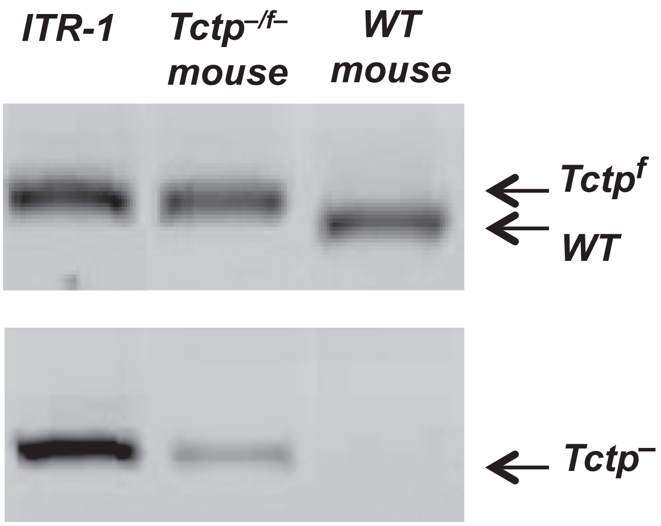

Figure EV2. *Tctp* genotyping of ITR-1 cells.

PCR signals of ITR-1 cells bearing one conditional allele (*Tctp* ^{f}) and one constitutive knockout allele (*Tctp* $^{-}$).

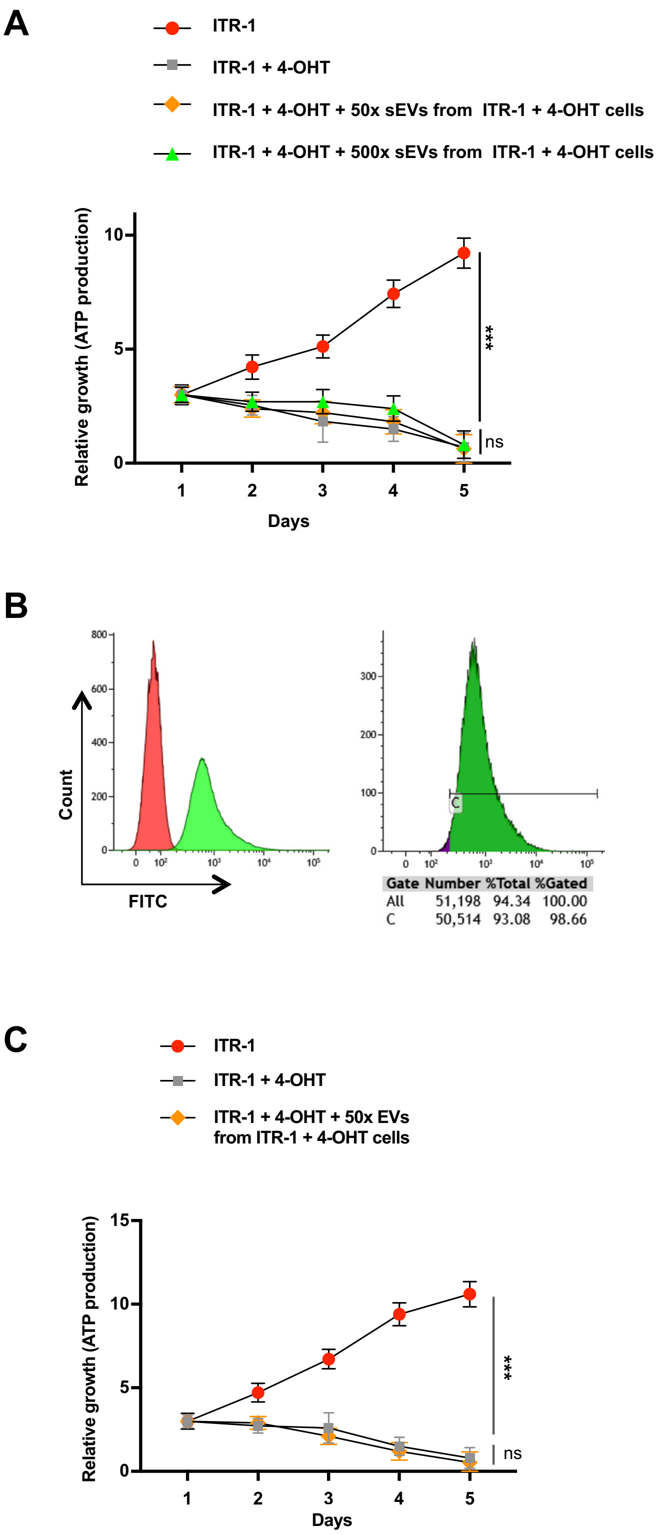
A
ITR-1
ITR-1 + 4-OHT
ITR-1 + 4-OHT + 50x sEVs from ITR-1 + 4-OHT cells
ITR-1 + 4-OHT + 500x sEVs from ITR-1 + 4-OHT cells
Relative growth (ATP production)
10
5
0
1
2
3
4
5
Days
***
ns
B
Count
FITC
800
600
400
200
0
0
10²
10³
10⁴
10⁵
300
200
100
C
Gate Number %Total %Gated
All 51,198 94.34 100.00
C 50,514 93.08 98.66
C
ITR-1
ITR-1 + 4-OHT
ITR-1 + 4-OHT + 50x EVs
from ITR-1 + 4-OHT cells
Relative growth (ATP production)
15
10
5
0
1
2
3
4
5
Days
***
ns

 © The Author(s)

Figure EV3. Complementation experiments: effect of sEVs and EVs from 4-OHT treated cells.

(**A**) Effect of sEVs from 4-OHT treated cells on the growth of 4-OHT treated cells. ITR-1 cells (red), ITR-1 cells treated with 4-OHT (gray). 4-OHT treated ITR-1 cells complemented with two concentrations of sEVs originating from 4-OHT treated ITR-1 cells (17×10^7 sEVs were added to 20,000 cells, 50x excess) (orange) or 170×10^7 sEVs, 500x excess (green) (independent biological replicates $n = 3$). (**B**) Representative experiment showing the uptake of FITC labeled sEVs derived from 4-OHT treated ITR-1 cells by 4-OHT treated ITR-1 cells. Left graph: Unlabeled 4-OHT treated ITR-1 cells alone (red), uptake of FITC labeled sEVs from 4-OHT treated ITR-1 cells by 4-OHT treated ITR-1 cells (green). Right graph: FITC negative population (purple), FITC positive gated population (green) (C gate). The value of the gating is displayed below the graph. (**C**) Effect of EVs (sEVs + NVs) from 4-OHT treated cells on the growth of 4-OHT treated cells. ITR-1 cells (red), ITR-1 cells treated with 4-OHT (gray). 4-OHT treated ITR-1 cells complemented with 50x excess of EVs originating from EVs from 4-OHT treated ITR-1 cells (orange) (17×10^7 EVs were added to 20,000 cells) (independent biological replicates $n = 4$). Data information: Statistical analysis was performed using Mann–Whitney test (**A**, **C**) Mean ± SEM. ns (not significant) ***$P < 0.001$. This control in vitro complementation experiment aims to assess the effect of sEVs, derived from 4-OHT treated ITR-1 cells, on cell growth. The conclusion of this experiment is that even a 500x excess of sEVs derived from 4-OHT treated ITR-1 cells is unable to restore tumor cell growth despite a highly efficient uptake of these sEVs. Source data are available online for this figure.

© The Author(s)

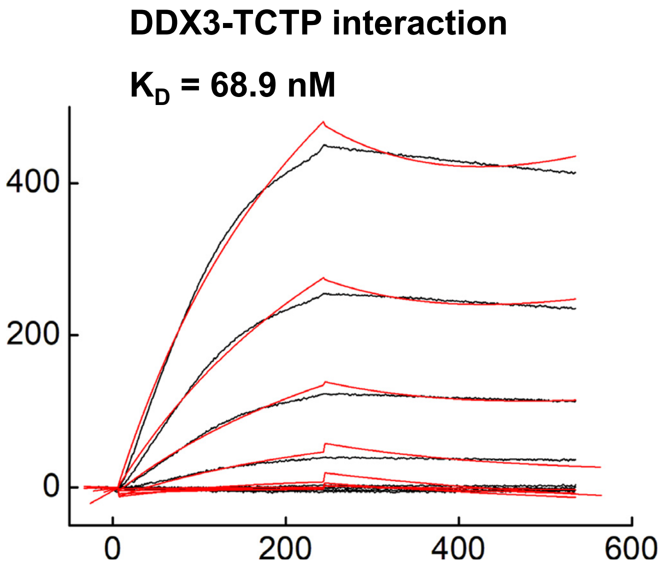

Figure EV4. Real-time interaction analysis of the binding of DDX3 to immobilized TCTP.

Real-time interaction profiles of the binding of DDX3 to immobilized TCTP. The experimental data are presented in black and the global analysis fit are in red. The concentration of DDX3 ranged from 100 nM to 0.171 nM. The measurements were performed at 25 °C. Source data are available online for this figure.

 © The Author(s)

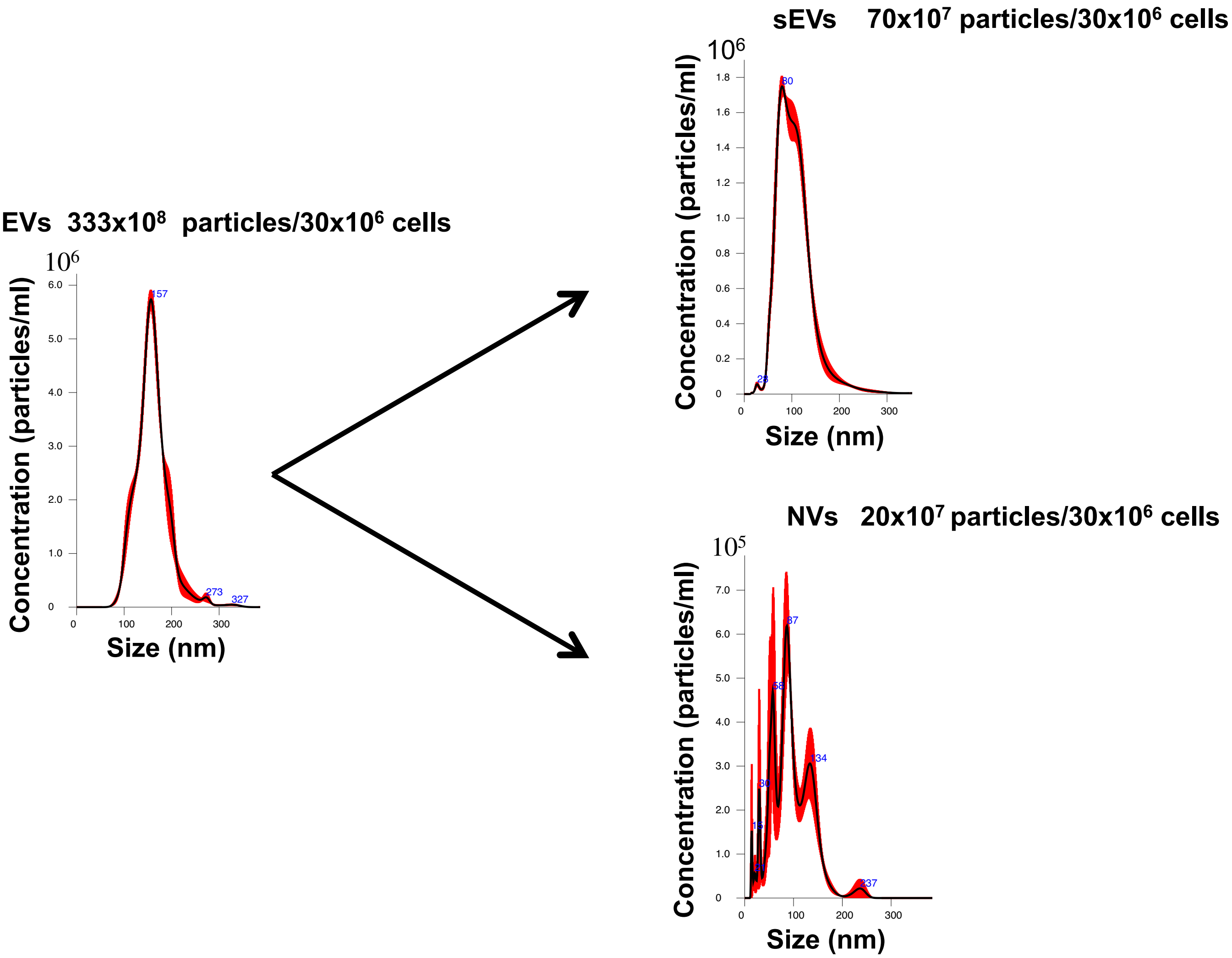

Figure EV5. Evaluation of sEV yield following high-resolution iodixanol gradient centrifugation.

The counts of EVs produced by 30×10^6 thymocytes (mean $= 253 \times 10^8$) (independent biological replicates $n = 4$) compared to the counts of sEVs (mean $= 72 \times 10^7$) (independent biological replicates $n = 8$) and NVs (16×10^7 mean) (independent biological replicates $n = 6$) indicate a loss of 57.5 fold $\{(253 \times 10^8/(72\ 10^7 + 16 \times 10^7)\}$ of sEVs together with NVs. Each sEV sample was diluted for an initial count to comply with the precision of the Nanosight measurements ranging from 10^6 to 10^{10}, being the most reliable window. Hence all the values presented take into account these dilutions. This 28.75 fold "loss" or more accurately "cleaning" using high-resolution iodixanol centrifugation remains so far one of the most precise methods to obtain purified sEVs. An entire body of literature shows that repeated steps of ultracentrifugation would also partially damage the exosomes and result in an important loss of particles. For these reasons, in the experiments using sEVs in vitro and in vivo, we used an excess of 50x of sEVs whether derived from thymocytes or cell lines.

© The Author(s)
